# AMPK activation reverts mouse epiblast stem cells to naive state

**DOI:** 10.1016/j.isci.2021.102783

**Published:** 2021-06-25

**Authors:** Yajing Liu, Junko Yamane, Akito Tanaka, Wataru Fujibuchi, Jun K. Yamashita

**Affiliations:** 1 The Department of Cell Growth and Differentiation, Center for iPS Cell Research and Application (CiRA), Kyoto University, Kyoto 606-8507, Japan; 2 The Department of Life Science Frontiers, Center for iPS Cell Research and Application (CiRA), Kyoto University, Kyoto 606-8507, Japan; 3 The Department of Animal Research Facility, Center for iPS Cell Research and Application (CiRA), Kyoto University, Kyoto 606-8507, Japan

**Keywords:** Cell biology, Stem cells research, Transcriptomics

## Abstract

Despite increasing knowledge on primed and naive pluripotency, the cell signaling that regulates the pluripotency type in stem cells remains not fully understood. Here we show that AMP kinase (AMPK) activators can induce the reversion of primed mouse epiblast stem cells (mEpiSCs) to the naive pluripotent state. The addition of AMPK activators alone or together with leukemia inhibitory factor to primed mEpiSCs induced the appearance of naive-like cells. After passaging in naive culture conditions, the colony morphology, protein expression, and global gene expression profiles indicated the naive state, as did germline transmission ability. Loss-of-function and gain-of-function studies suggested that p38 is a critical downstream target in AMPK activation. Finally, single-cell RNA sequencing analysis revealed that the reversion process through AMPK signaling passes an intermediate naive-like population. In conclusion, the AMPK pathway is a critical driving force in the reversion of primed to naive pluripotency.

## Highlights

• AMPK activation can induce mouse primed stem cell reversion to naive state
• Reverted cells fully obtain naive pluripotency
• p38 is a critical downstream molecule of AMPK pathway in the reversion process
• Dppa5 and Dazl are highly expressed in intermediate reversion cells

## Graphical abstract

**Figure:**
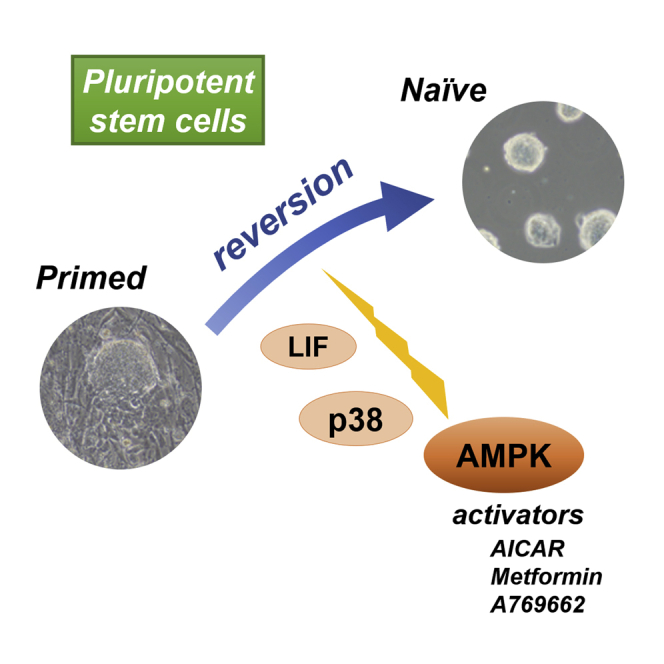

## Introduction

Studies of mouse embryonic development and stem cells have shown that there are at least two pluripotent states: naive and primed (Nichols and Smith, 2009, 2012; Kalkan and Smith, 2014; Martello and Smith, 2014). Mouse embryonic stem cells (mESCs), which are derived from inner cell mass at embryonic day (E) 3.5–4.5, are thought to be similar to naive pre-implantation epiblast (Martin, 1981; Evans and Kaufman, 1981; Boroviak et al., 2014, 2015). Naive mESCs can be fully maintained *in vitro* by culturing them in two small molecule inhibitors (2i) of kinases (MEK and GSK3) and leukemia inhibitory factor (LIF)(2iL) (Williams et al., 1988; Ying et al., 2008). Naive mESCs show small dome-shaped colonies, express specific naive pluripotent markers, exhibit unique global gene expression profiles and epigenetic modifications, and contribute to the chimera component after blastocyst injection including germline transmission (Nichols and Smith, 2009; Blair et al., 2011; Marks et al., 2012; Martello and Smith, 2014).

In contrast to naive mESCs, mouse epiblast stem cells (mEpiSC) derived from postimplantation embryonic epiblast at E5.5–6.5 show primed features. These cells can be maintained by fibroblast growth factor-2 (FGF2) and Activin A signaling but differentiate or die in 2iL condition. Furthermore, they are characterized by large flat cup-shaped colonies and pluripotency gene expressions that are distinct from naive cells and also are far less efficient at chimera contribution (Nichols and Smith, 2009; Blair et al., 2011; Brons et al., 2007; Tesar et al., 2007; Hanna et al., 2009a).

Primed mEpiSCs can be reverted to naive mESCs in many ways, including LIF-STAT3 pathway activation (Bao et al., 2009); gene transfection with Klf4 (Guo et al., 2009; Hanna et al., 2009b), c-Myc (Hanna et al., 2009b), Klf2 (Hall et al., 2009), Nanog (Silva et al., 2009; Greber et al., 2010), or the nuclear receptor genes Nr5a1/2 (Guo and Smith, 2010), Prdm14 (Gillich et al., 2012), and Gbx2 (Tai and Ying., 2013); and small molecule treatment, including parnate and inhibitors of ALK4/5/7, MEK, FGFR, and GSK3 (Zhou et al., 2010) or casein kinase 1 alpha (CK1alpha) (Illich et al., 2016). Currently, 2iL condition is considered sufficient at maintaining the naive state but insufficient at reverting EpiSCs to naive cells. Which signals are the most critical for resetting primed pluripotency to naive pluripotency remains unclear.

We previously revealed that the activation of adenosine monophosphate (AMP)-activated protein kinase (AMPK) contributes to the maintenance of naive mESCs, implying the involvement of the AMPK pathway in mediating naive pluripotency (Liu and Yamashita, 2019). Additionally, the AMPK signal has been shown to promote the reprogramming process from somatic cells to pluripotent stem cells (Ma et al., 2015). Nevertheless, whether the AMPK signal can revert primed cells to naive state is unclear. Although AMPK knockout experiments have been done to study energy homeostasis at the cellular and whole body level (Viollet et al., 2009; Hardie, 2014; Stauffer et al., 2019), the role of AMPK in naive reversion or reprogramming has not been suggested.

In the present study, we show that three AMPK activators, AICAR (5-aminoimidazole-4-carboxamide ribonucleotide), A769662, and metformin, can revert primed mEpiSCs to naive mESCs. Even in the absence of 2iL, AICAR treatment alone induced the appearance of naive-like cells from primed mEpiSCs. The reverted cells satisfied the criteria of naive mESCs: they were maintained and grown in 2iL condition, showed naive gene and protein expression patterns, and were capable of chimera formation and germline transmission. Furthermore, we identified p38 as a candidate functional downstream in the AMPK-elicited reversion process. Finally, single-cell RNA sequencing (RNA-seq) analysis during the reversion process demonstrated the appearance of an intermediate naive-like population marked by the high mRNA expression of Dppa5a and Dazl, both of which were reported to be involved in naive pluripotency (Qian et al., 2016; Kilens et al., 2018; Welling et al., 2015). Our data indicate that AMPK signaling reverts primed mEpiSCs to the naive state, providing new insights into the molecular switch mediating the transition between different pluripotency states.

## Results

### AMPK activators induce reversion of primed mEpiSCs to naive-like cells

**Figure 1:**
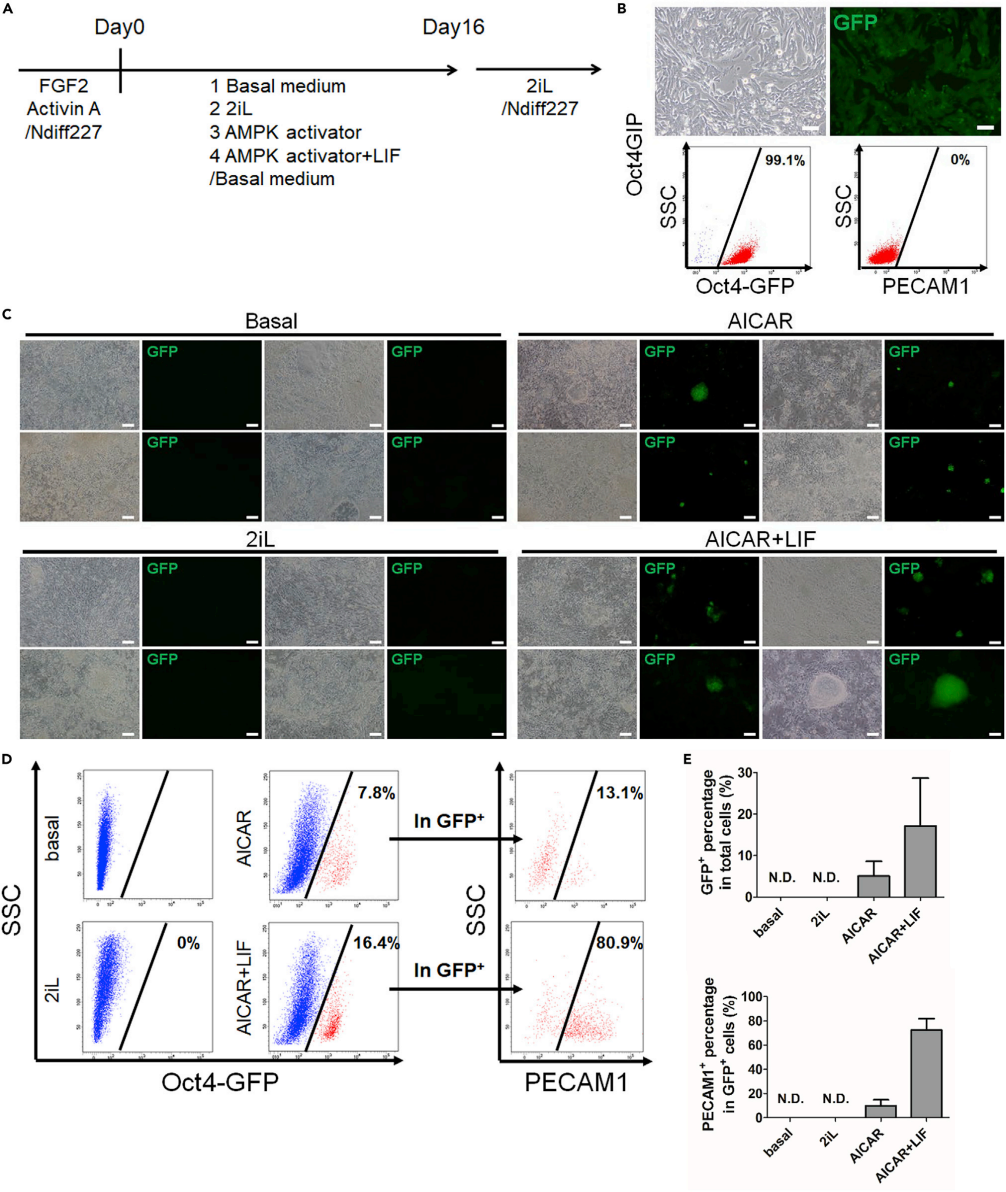
AICAR induces reversion of primed mEpiSCs to naive-like cells (A) Schema of the chemical reversion protocol. (B) Maintained mouse primed epiblast stem cells (mEpiSCs: Oct4GIP) in FGF2 and Activin. (A) Upper panels: cell morphology and Oct4-GFP expression. Lower panels: FACS analysis for Oct4-GFP and PECAM1. SSC, side scatter. Scale bars, 200 μm. (C) Cell morphology and Oct4-GFP expression of Oct4GIP after treatment with Basal medium alone, 2i/L, AICAR, or AICAR + LIF for 16 days. Four pairs of phase contrast and Oct4-GFP images are shown for each condition. Scale bars, 200 μm. (D) FACS analysis for Oct4-GFP and PECAM1 expression after 16 days of reversion. Red, Oct4-GFP-positive fraction. Oct4-GFP-positive percentages in total cells (middle panels) and PECAM1-positive percentages in the Oct4-GFP-postive population (right panels) are indicated. (E) Quantitative evaluation of Oct4-GFP-positive cells in total cells and PECAM1-positive cells in Oct4-GFP-positive cells (mean ± SD, n = 5; N.D., not detected). See also Figures S1 and S2.

Previously, we found that AMPK activators contribute to the maintenance of naive mESCs in the absence of 2iL with increased expressions of the naive pluripotent gene network (Liu and Yamashita, 2019). This prompted us to examine whether activation of the AMPK pathway can not only maintain the naive state but also revert primed cells to the naive state. Thus, we tested the effect of AMPK activators on the reversion of primed mEpiSCs. We employed an mEpiSC line, Oct4GIP, which carries an Oct4 promoter/enhancer-driven eGFPiresPuro transgene (Oct4-GFP) expressed in primed and naive pluripotent stem cells (Guo et al., 2009; Wray et al., 2010). We cultured Oct4GIP cells in Basal medium (a basic medium for mESC maintenance; see STAR Methods) together with various combinations of reagents (Figure 1A). Before changing to the medium for reversion, Oct4GIP cells were maintained in the mEpiSC culture condition, which contained serum-free medium Ndiff 227 supplemented with FGF2 and Activin A (Guo et al., 2009). Almost all cells (99.2% ± 0.7%) were positive for Oct4-GFP but negative for the naive ESC marker PECAM1/CD31 (Illich et al., 2016), indicating that the cells were in the primed state (day 0; d0) (Figure 1B). The reversion process at d4, d8, and d12 showed that when the cells were cultured in Basal medium alone or Basal medium with 2iL (naive maintenance condition), GFP expression disappeared within 4 days and all cells showed a differentiated morphology (Figure S1A), resulting in no GFP expression on d16 (Figure 1C). On the other hand, when the cells were cultured with an AMPK activator, AICAR, or AICAR+LIF, the GFP expression was largely decreased until d8 and totally disappeared around d10 to d12 (Figure S1A). Some GFP^+^ colonies re-appeared around d16 (Figure 1C). Fluorescence-activated cell sorting (FACS) analysis showed approximately 5% of total cells (5.34% ± 3.27%) expressed Oct4-GFP (Figures 1D and 1E) on d16 by culturing with AICAR alone. In the AICAR+LIF condition, approximately 17% of total cells (17.1% ± 11.6%) were OCT4-GFP positive. We confirmed the appearance of PECAM1/CD31 in Oct4-GFP-positive cells. Among OCT4-GFP^+^ cells, approximately 9% (9.04% ± 3.88%) in AICAR-alone condition and 72% (72.6% ± 9.23%) in AICAR and LIF condition, respectively, were positive for PECAM1 (Figures 1D and 1E). The appearance of OCT4 and PECAM1 double-positive cells suggested that AICAR treatment can revert primed mEpiSCs to the naive state. Alkaline phosphatase (AP) staining on d16 showed that AP-positive colonies were observed with AICAR treatment alone but more AP-positive colonies were formed with AICAR+LIF (Figure S1B). Immunostaining for colonies on d16 also confirmed the appearance of Oct4^+^PECAM1^+^ cells and Oct4^+^PECAM1^-^ cells. We further checked another naive marker, Klf4, finding it was co-expressed in Oct4^+^PECAM1^+^ cells but not in Oct4^+^PECAM1^-^ cells, suggesting that Oct4^+^PECAM1^+^ cells were reverted to the naive state successfully but Oct4^+^PECAM1^-^ cells were not (Figure S1C).

We also tested two other AMPK activators, A769662 and metformin (Figure S2A). Similar to the AICAR treatments, Oct4-GFP^+^ cells became fewer and disappeared by d12 but reemerged by around d16 after treatment with A769662 alone and A769662 or metformin with LIF (Figures S2B and S2C). FACS analysis showed that in all three conditions, Oct4-GFP and PECAM1 double-positive cells were fewer than in the AICAR treatments described earlier, but they were nevertheless present, indicating that several AMPK activators can revert primed mEpiSCs (Figures S2D and S2E). Taking our data together, AMPK activators can induce the reversion of primed mEpiSCs to the naive state, more effectively if mixed with LIF.

### Reverted cells show naive pluripotency features

**Figure 2:**
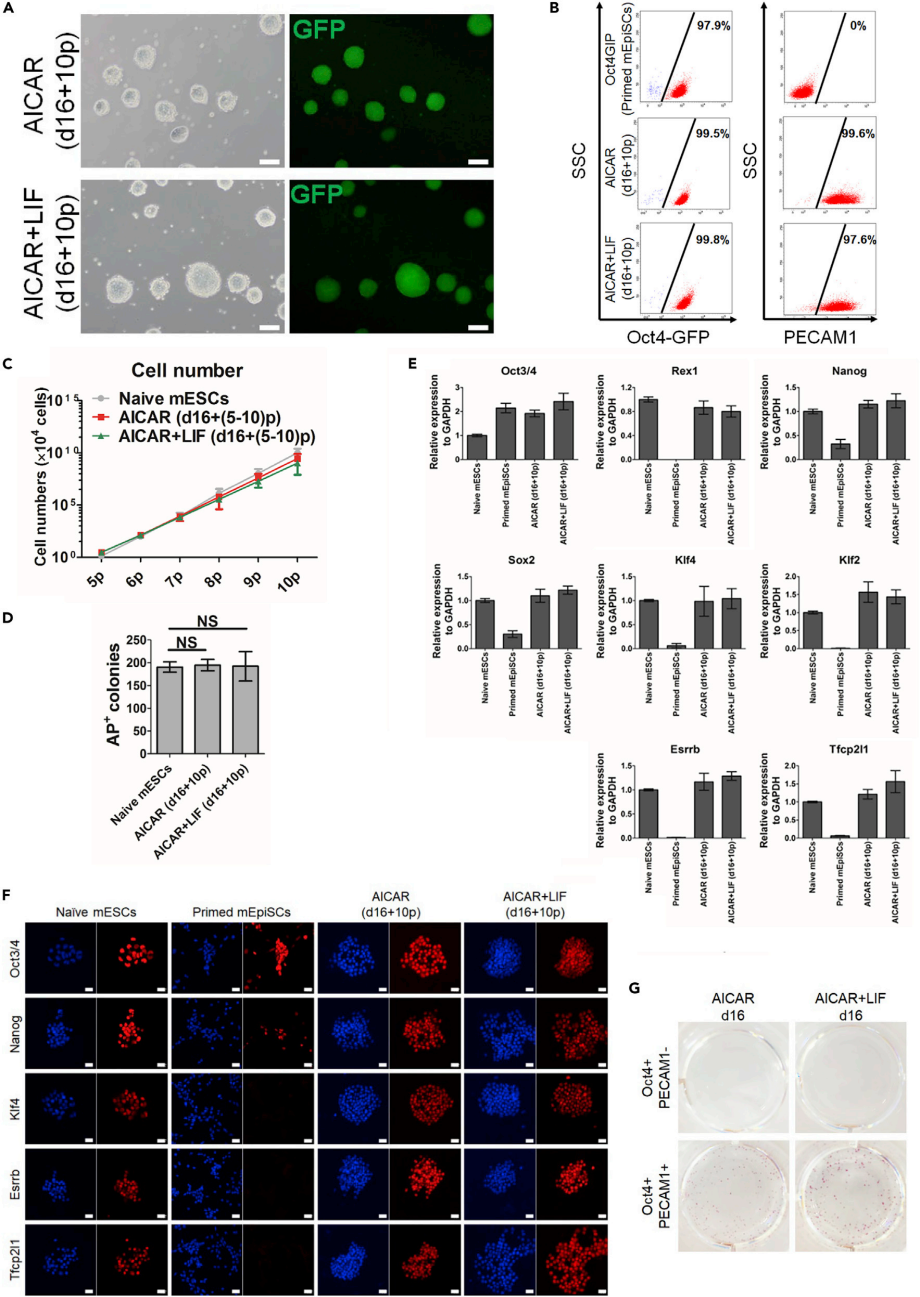
AICAR-reverted cells show naive pluripotency features (A) Cell morphology and Oct4-GFP expression in AICAR- and AICAR + LIF-reverted cells. After treatment with AICAR or AICAR + LIF in Basal medium for 16 days, cells were harvested and re-cultured in 2i/L condition for 10 passages (d16 + 10p). Scale bars, 200 μm. (B) FACS analysis for Oct4-GFP and PECAM1 in maintained primed mEpiSCs (Oct4GIP) and cells reverted by AICAR or AICAR + LIF (d16 + 10p). Percentages are Oct4-GFP-positive or PECAM1-positive cells in total cells. (C) Proliferation of reverted cells. Numbers of reverted cells during passages 5 to 10 compared with naive mESCs in 2i/L (mean ± SD; n = 5). (D) Alkaline phosphatase-positive (AP ^+^) colony formation assay. Five hundred reverted cells (d16 + 10p) or naive mESCs were plated in 2i/L condition. After 5 days of culture, AP^+^ colonies were counted (mean ± SD, n = 5, NS, not significant). (E) Naive and pluripotent gene mRNA expressions (qPCR) in naive mESCs, primed mEpiSCs (Oct4GIP), and cells reverted by AICAR or AICAR + LIF (d16 + 10p). Naive mESCs: Rex1-GFP cells maintained in 2iL. Expression levels are normalized to GAPDH. Data are represented as the mean ± SD (n = 5; with technical triplicates). The results of naive ESCs were set to 1. (F) Immunofluorescence staining for naive and pluripotent markers (red) in naive mESCs (Rex1-GFP), primed mEpiSCs (Oct4GIP), and cells reverted by AICAR or AICAR + LIF (d16 + 10p). DAPI (blue), nuclear staining. Scale bars, 20 μm. (G) AP-staining colony assay of Oct4^+^PECAM1^+^ and Oct4^+^PECAM1^-^ cells reverted by AICAR or AICAR + LIF. Oct4^+^PECAM1^+^ and Oct4^+^PECAM1^-^ cells were sorted after 16 days of AICAR or AICAR + LIF treatment, respectively. Then, 500 cells from each condition were plated in 2i/L condition. After 5 days of maintenance, AP staining was performed. See also Figure S3.

Next, we checked whether the naive-like cells that appeared from the treated primed mEpiSCs satisfied the criteria of naive mESCs. We changed the culture medium at d16, which was when Oct4-GFP^+^ cells had already appeared, from Basal medium with AMPK activators to Ndiff227 medium with 2iL to promote naive cell growth and maintenance. After 10 passages (d16+10p), compact and dome-shaped naive-like colonies with homogenous Oct4-GFP expression selectively grew in the 2iL condition (Figure 2A). FACS analysis showed that whereas primed mEpiSCs (Oct4GIP) were positive for OCT4-GFP but negative for PECAM1, naive-like cells induced with AICAR alone or AICAR with LIF (reverted cells, d16+10p) were largely homogenously positive for PECAM1 (Figure 2B). Those cells were maintained and grew well in 2iL condition by single-cell dissociation with similar expansion efficiency (Figure 2C) and showed comparable AP-positive colony formation with control naive mESCs (Figure 2D). According to a previous report, Oct4GIP cells die or differentiate after several passages in 2iL condition (Guo et al., 2009). We confirmed that Oct4GIP cells cultured in 2iL could not be maintained after seven passages even with mouse embryonic fibroblasts (MEFs), indicating that mEpiSCs never give rise to naive cells if simply cultured in 2iL condition (Figure S3A). The mRNA expressions of pluripotent genes showed that although Oct4 was comparably expressed in naive mESCs, primed mEpiSCs, and reverted cells, many naive-specific genes, such as Rex1, Klf4, Klf2, Esrrb, and Tfcp2l1, were specifically expressed only in the reverted cells and naive mESCs (Figure 2E). Furthermore, naive-specific proteins were clearly and homogenously expressed in the nuclei of the reverted cells, consistent with naive mESCs but not primed mEpiSCs (Figure 2F). AP-positive colony formation indicated that Oct4^+^PECAM1^+^ cells sorted on d16 formed colonies in 2iL but Oct4^+^PECAM1^-^ did not, verifying that Oct4^+^PECAM1^+^ cells were successfully reverted to the naive state, but Oct4^+^PECAM1^-^ cells were not (Figure 2G). We further evaluated naive-like cells induced with other AMPK activators. Similar to those induced with AICAR, compact and dome-shaped naive-like colonies with homogenous Oct4-GFP expression selectively appeared from Oct4-GFP^+^ cells induced with A769662 alone, A769662+LIF, or metformin+LIF treatment (d16+10p) (Figure S3B). FACS analysis showed that the reverted cells induced in these three conditions were largely homogeneously positive for PECAM1 (Figure S3C). Additionally, they were maintained and expanded well in 2iL condition (Figure S3D) and showed comparable AP-positive colony formation with control naive mESCs (Figure S3E). Consistently, many naive-specific mRNAs and proteins were expressed at comparable levels as in naive mESCs (Figures S3F and S3G).

**Figure 3:**
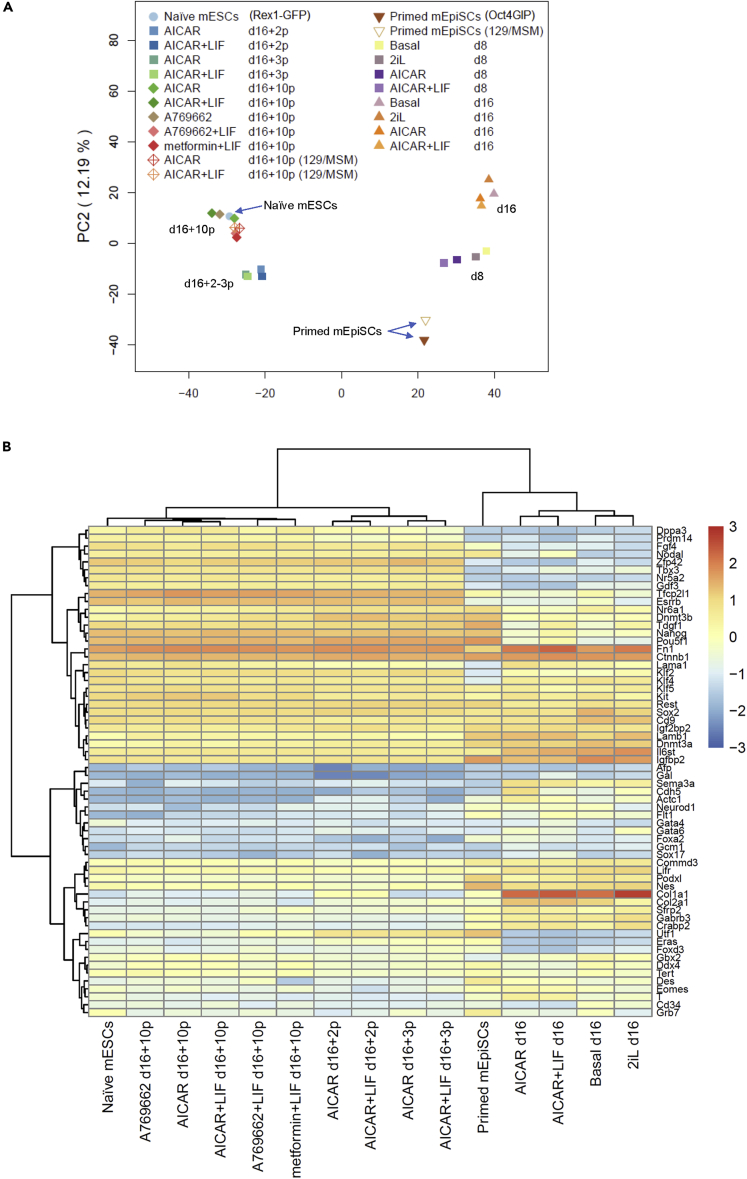
RNA-seq analysis during the reversion process (A) PCA of different cell types by PCF gene signatures (2,036 genes). Naive mESCs: Rex1-GFP cells; primed mEpiSCs: Oct4GIP cells and 129/MSM; and total cells reverted by different AMPK activators cultured in 2iL condition after several days (d) and passages (p). (B) Heatmap of gene expressions for pluripotency regulators and lineage markers revealed two major groups: naive-like populations shown in left 10 lanes, and primed-like populations shown in right 5 lanes. Red and blue colors indicate relatively high and low gene expression levels, respectively. See also Figure S4.

We further evaluated the global gene expression of the AMPK-induced reverted cells by RNA-seq. Principal-component analysis (PCA) of 34,489 genes showed that reverted cells were closely similar to naive mESCs (Figure S4). Furthermore, the identified PCF (pluripotent cell fate) gene signature was reported to reveal a better separation of naive and primed pluripotent cells than global transcripts (Fidalgo et al., 2016). PCA using 2,036 PCF genes successfully depicted the naive and primed cells. After expanding AMPK activator-induced cells with several passages in 2iL condition (d16+2p, 3p, 10p), all reverted cells belonged to a cluster near naive mESCs and were mutually exclusive from the clusters of primed mEpiSCs and AMPK activator-treated cells before expansion with 2iL condition (d8 and d16) (Figure 3A). The first principal component (PC1) captured much of the variation, indicating a clear correspondence of reverted cells to naive cells but not primed ones (Figure 3A). Reverted cells showed a short distance when cells were passaged only two or three times (d16+2p, 3p) in 2iL condition but almost coincided with naive mESCs after 10 passages (d16+10p), indicating that the reverted cells could fully obtain naive properties after enough passages in 2iL condition. Even though d16 cells included some naive-like Oct4-GFP and PECAM1 double-positive cells (Figure 1D, 1E, S1B, and S1C), global gene expression patterns were not reflected in these cells, possibly because their population was small before passaging and expanding in 2iL condition. PC1 largely depicted major populations until d16 that were differentiating and separating from the naive and primed stem cell populations. The heatmap of the mRNA expression chosen from another panel of genes for pluripotency regulators and lineage markers (Takashima et al., 2014) revealed that the naive-like cells induced by different AMPK activators shared a similar expression pattern with naive mESCs and different from that of primed mEpiSCs (Figure 3B). For example, naive markers Esrrb, Zfp42 (also known as Rex1), Nr5a2, Tfcp2l1, and Klf2 were expressed in reverted cells at levels comparable to those in naive mESCs. In contrast, lineage markers such as Desmin, Eomoesdermin, T, and Foxa2 were lower in reverted cells than in primed mEpiSCs. In addition to Oct4GIP mEpiSCs, we also examined another mEpiSC line, 129/MSM, obtained from mating male MSM/Ms mice and female 129X1/SvJ mice (Yagi et al., 2019). Both the PCA and heatmap clearly showed that 129/MSM mEpiSCs were reverted into naive the state (Figures 3 and S4). Thus, AMPK activators can revert cells into a state that satisfies the *in vitro* criteria of naive mESCs.

### Reverted cells contribute to chimera formation and germline transmission

**Figure 4:**
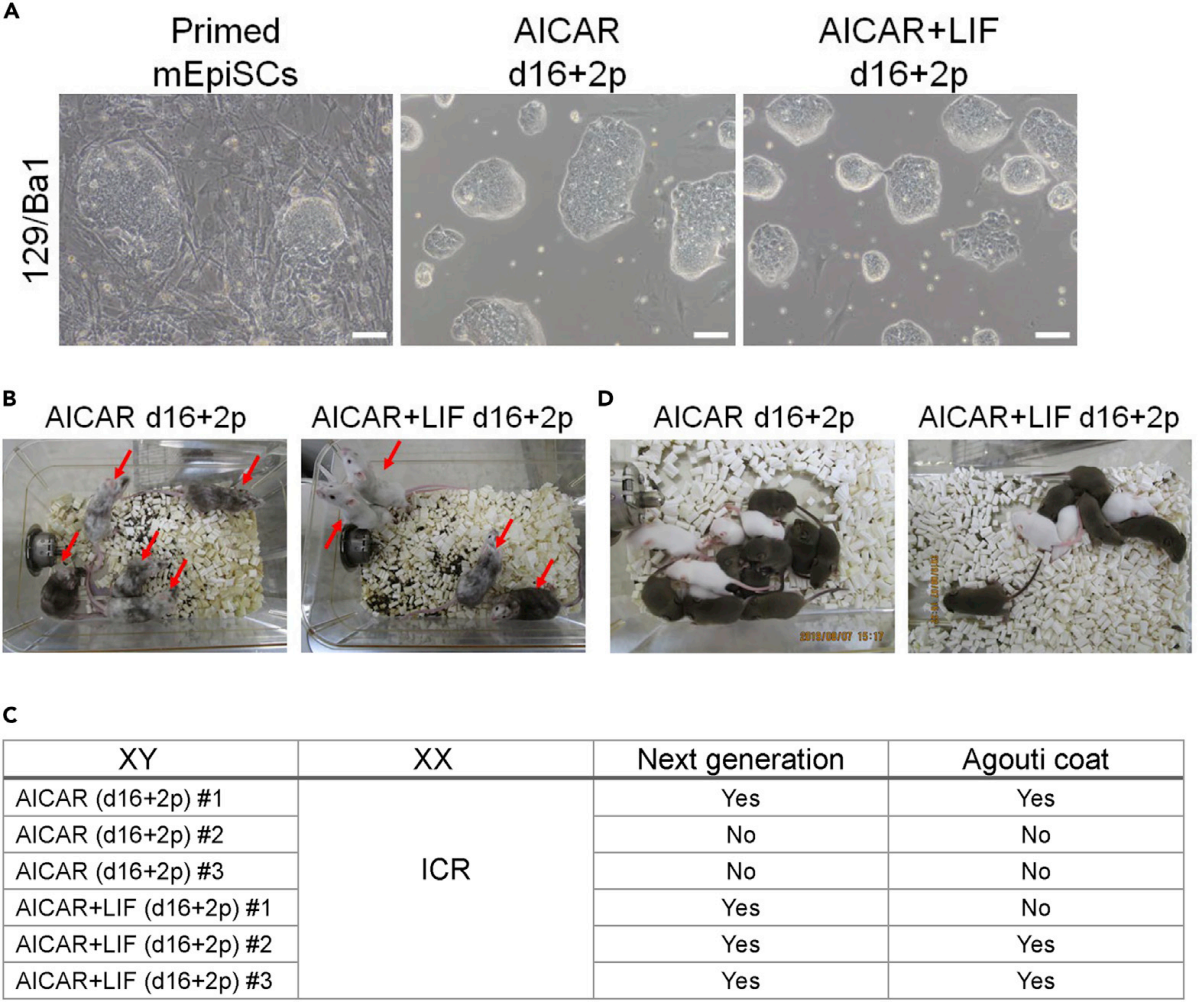
Reverted cells contribute to chimera formation and germline transmission (A) Cell morphology of the primed mEpiSC line 129/Ba1 and 129/Ba1 reverted by AICAR or AICAR + LIF (d16+2p). Scale bars, 200 μm. (B) Chimera mice after blastocyst injection. 129/Ba1 cells reverted by AICAR or AICAR + LIF after expansion in 2iL were injected into female ICR blastocysts. Red arrows indicate chimera mice. (C) Germline transmission. Male chimera mice were mated with wild-type female ICR mice. The summary of the mating results is shown. (D) Representative photographs of mice with the germline transmission. Agouti mice indicate germline transmission.

Next, we confirmed the naive status of AMPK-induced cells more definitively by verifying the contribution to chimera formation and germline transmission. For these experiments, we used two primed mEpiSC lines, 129/Ba1 obtained from 129/Sv×C57BL/6N mice (Sugimoto et al., 2015) and 129/MSM. After treatment with AICAR alone or AICAR+LIF followed by maintenance and several passages in 2iL, 129/Ba1cells showed the naive-like colony morphology similar to Oct4GIP cells (Figure 4A). Naive-like reverted 129/Ba1 cells were injected into blastocysts of female ICR mice and examined for their contribution of chimera formation. The majority of pups showed successful coat color chimerism (Figure 4B). After mating male chimera mice with female ICR mice, agouti-coated mice were produced, indicating germline transmission of the 129/Ba1 cell genome (Figures 4C and 4D). In 129/MSM cells, the cell morphology and AP staining marking naive-like cells were successfully obtained (Figures S3H and S3I) and chimera formation was observed (data not shown). All these results indicate that AMPK activation can induce the reversion of primed mEpiSCs to naive mESCs.

The reversion efficiencies of the AMPK activators in several cell lines are summarized in Table 1. We defined success of the reversion as naive-like colonies obtained from primed mEpiSCs maintained in 2iL condition. Notably, AMPK activator alone (AICAR or A769662) induced the appearance of naive-like cells from primed mEpiSCs. The addition of LIF enhanced the efficiency of the reversion, and in the case of AICAR, the efficiency reached 100% in all primed mEpiSCs examined (Oct4GIP, 129/Ba1, and 129/MSM). All these results indicate that AMPK activation can drive the reversion of primed mEpiSCs to the naive pluripotent state.

**Table 1:** Success rates of mEpiSC reversion by AMPK activators or p38 activation

Cell line	Reversion condition	Success times/total times	Success rate (%)
Oct4GIP	Basal	0/9	0
Oct4GIP	2iL	0/21	0
Oct4GIP	AICAR	5/10	50
Oct4GIP	AICAR + LIF	10/10	100
129/Ba1	Basal	0/2	0
129/Ba1	2iL	0/2	0
129/Ba1	AICAR	1/2	50
129/Ba1	AICAR + LIF	2/2	100
129/MSM	Basal	0/4	0
129/MSM	2iL	0/4	0
129/MSM	AICAR	3/5	60
129/MSM	AICAR + LIF	5/5	100
Total	Basal	0/15	0
	2iL	0/27	0
	AICAR	9/17	53
	AICAR + LIF	17/17	100
Oct4GIP	A769662	4/7	57
Oct4GIP	A769662+LIF	5/7	71.4
Oct4GIP	Metformin + LIF	5/7	71.4
Oct4GIP (ca-p38)	dox-	0/9	0
Oct4GIP (ca-p38)	LIF + dox-	0/9	0
Oct4GIP (ca-p38)	dox+	1/9	11.1
Oct4GIP (ca-p38)	LIF + dox+	3/9	33.3

### p38 is a critical downstream target in AMPK activator-elicited reversion

**Figure 5:**
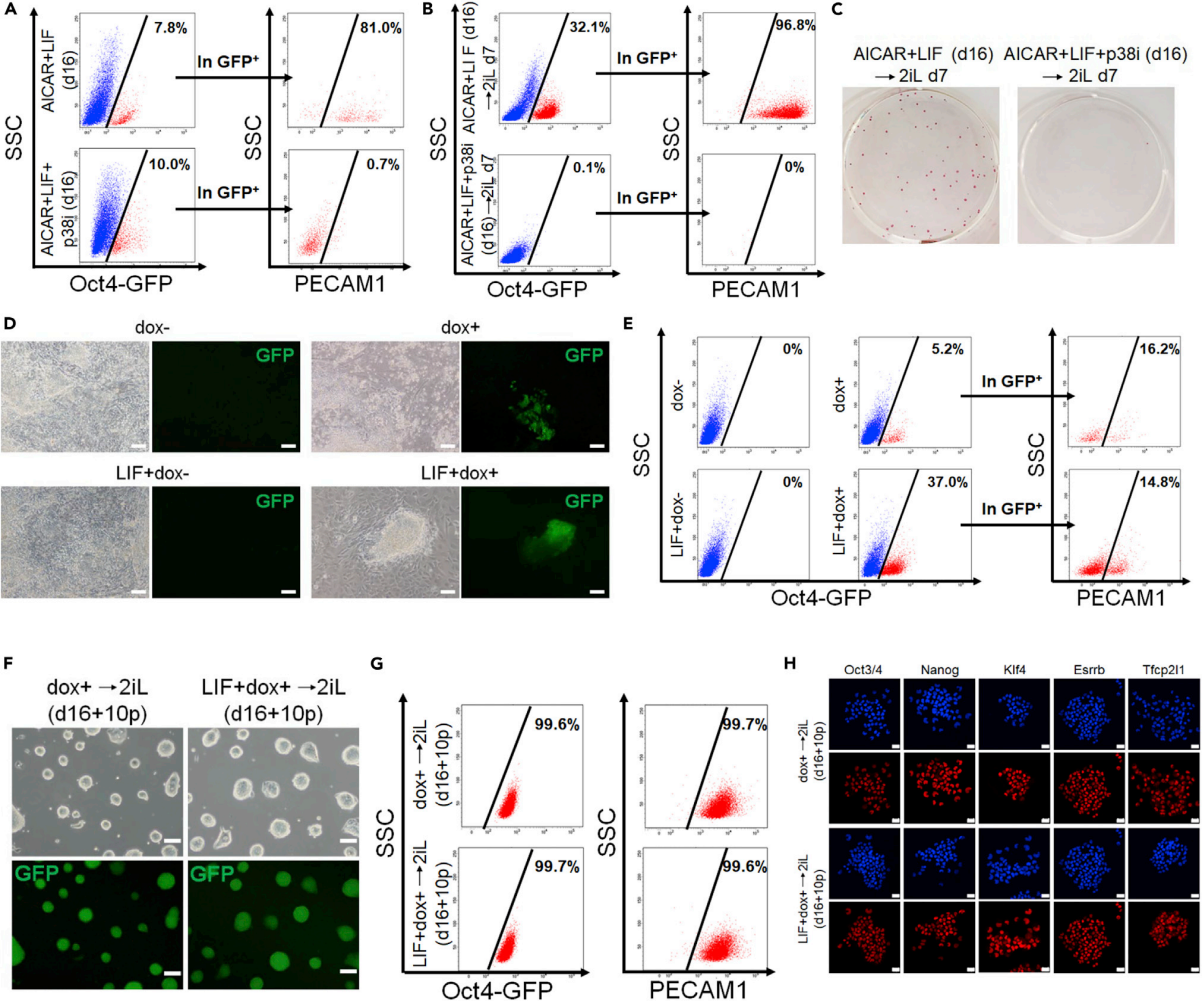
p38 is a critical downstream target in AMPK activator-elicited reversion (A–C) p38 inhibition perturbed the AICAR-induced reversion of primed mEpiSCs. (A) FACS analysis of AICAR + LIF and of AICAR + LIF + p38 inhibitor (p38i; SB203580 [10 μM])-treated cells (d16). Red represents the Oct4-GFP-positive fraction. Oct4-GFP-positive percentages in total cells (left panels) and PECAM1-positive percentages in the Oct4-GFP-postive population (right panels) are indicated. (B) FACS analysis of bulk cells cultured in 2iL for 7 days after reversion by AICAR + LIF or AICAR + LIF + p38i. Red represents the Oct4-GFP-positive fraction. Oct4-GFP-positive percentages in total cells (left panels) and PECAM1-positive percentages in the Oct4-GFP-postive population (right panels) are indicated. (C) AP staining of bulk cells cultured in 2iL for 7 days after reversion by AICAR + LIF or AICAR + LIF + p38i. (D–H) Reversion of primed mEpiSCs by p38 activation. A constitutively active form of p38 (CA-p38) was introduced into Oct4GIP and expressed with the dox-inducible system (dox+). (D) Representative cell morphology and Oct4-GFP expression after 16 days (d16) of dox (1 μg/mL) and/or LIF treatment. Scale bars, 200 μm. (E) FACS analysis for Oct4-GFP and PECAM1 expresssion after 16 days (d16) of dox and/or LIF treatment. Red represents the Oct4-GFP-positive fraction. Oct4-GFP-positive percentages in total cells (left panels) and PECAM1-positive percentages in the Oct4-GFP-postive population (right panels) are indicated. (F) Representative cell morphology and Oct4-GFP expression in cells reverted with dox+ or LIF+ dox+ condition after expansion in the 2iL condition (d16 + 10p). Scale bars, 200 μm. (G) FACS analysis for Oct4-GFP and PECAM1 in cells reverted with dox+ or LIF+ dox+ (d16 + 10p). Percentages are Oct4-GFP-positive or PECAM1-positive cells in total cells. (H) Immunofluorescence staining for naive and pluripotent markers (red) in cells reverted with dox+ or LIF+ dox+ (d16 + 10p). DAPI (blue), nuclear staining. Scale bars, 20 μm. See also Figure S5.

Next, we examined the molecular mechanisms of the AMPK activator-induced reversion to naive cells. We previously demonstrated that p38 is functional downstream of the AMPK signal to maintain naive pluripotency (Liu and Yamashita, 2019). The activation of p38 was also reported to promote the reprogramming of somatic cells to pluripotent stem cells (Xu et al., 2013). Thus, we hypothesized that p38 functions downstream of the AMPK signal during the reversion process. AMPK activators increased the phosphorylation of p38 compared with control cells (Figure S5A). We examined the effects of p38 inhibition on the AICAR + LIF-induced reversion to the naive state. Although the appearance of OCT4-GFP^+^ cells was similar, PECAM1 expression in OCT4-GFP^+^ cells was largely reduced by a p38 inhibitor (p38i), SB203580, at d16 (Figure 5A). Seven days after changing the medium to 2iL condition, we observed that whereas Oct4-GFP^+^/PECAM1^+^ cells began to grow from the AICAR + LIF-induced cells, Oct4-GFP^+^/PECAM1^+^ cells never appeared from AICAR + LIF + p38i-treated cells (Figure 5B). AP staining, which is negative when primed mEpiSCs are cultured in 2iL condition (Martello et al., 2012), was negative in AICAR + Lif + p38i-treated cells (Figure 5C). The appearance of naive-like cells elicited by A769662 + LIF or metformin + LIF was similarly blocked by SB203580 (Figures S5B and S5C). p38i did not affect the total cell number of AICAR + LIF-treated cells (d16) (Figure S5D) or naive mESCs (Figure S5E), suggesting that it blocks the naive conversion process but not cell expansion. These results indicated that p38 signaling is critical for the AMPK activation of the naive reversion process.

We also conducted p38 gain-of-function experiments. We generated an Oct4GIP cell line carrying a tetracycline-inducible (Tet-ON) constitutively active form of p38 that contains D176A and F327S mutations (Xu et al., 2013; Diskin et al., 2004) and activated the p38 pathway with doxycycline (dox) treatment (Figure S5F). p38 activation alone or with LIF induced the appearance of Oct4-GFP^+^ colonies after 16 days in Basal medium (Figure 5D). Among those Oct4-GFP^+^ cells, PECAM1^+^ cells were observed (Figure 5E). Then the medium was changed to 2iL condition, and the cells were passaged several times. Similar to the AMPK activator treatment experiments, homogeneous Oct4-GFP^+^ naive-like colonies appeared even with p38 activation alone (dox+) (Figure 5F). These cells were uniformly Oct4-GFP and PECAM1 double positive (Figure 5G) and showed clear naive marker protein expression (Figure 5H). As summarized in Table 1, the reversion efficiency with p38 activation was not as good as with AMPK activators, suggesting that p38 is critical but not sufficient for the AMPK-mediated reversion to the naive state.

### Single-cell RNA-seq transcriptome profiles of primed stem cell reversion to the naive state

**Figure 6:**
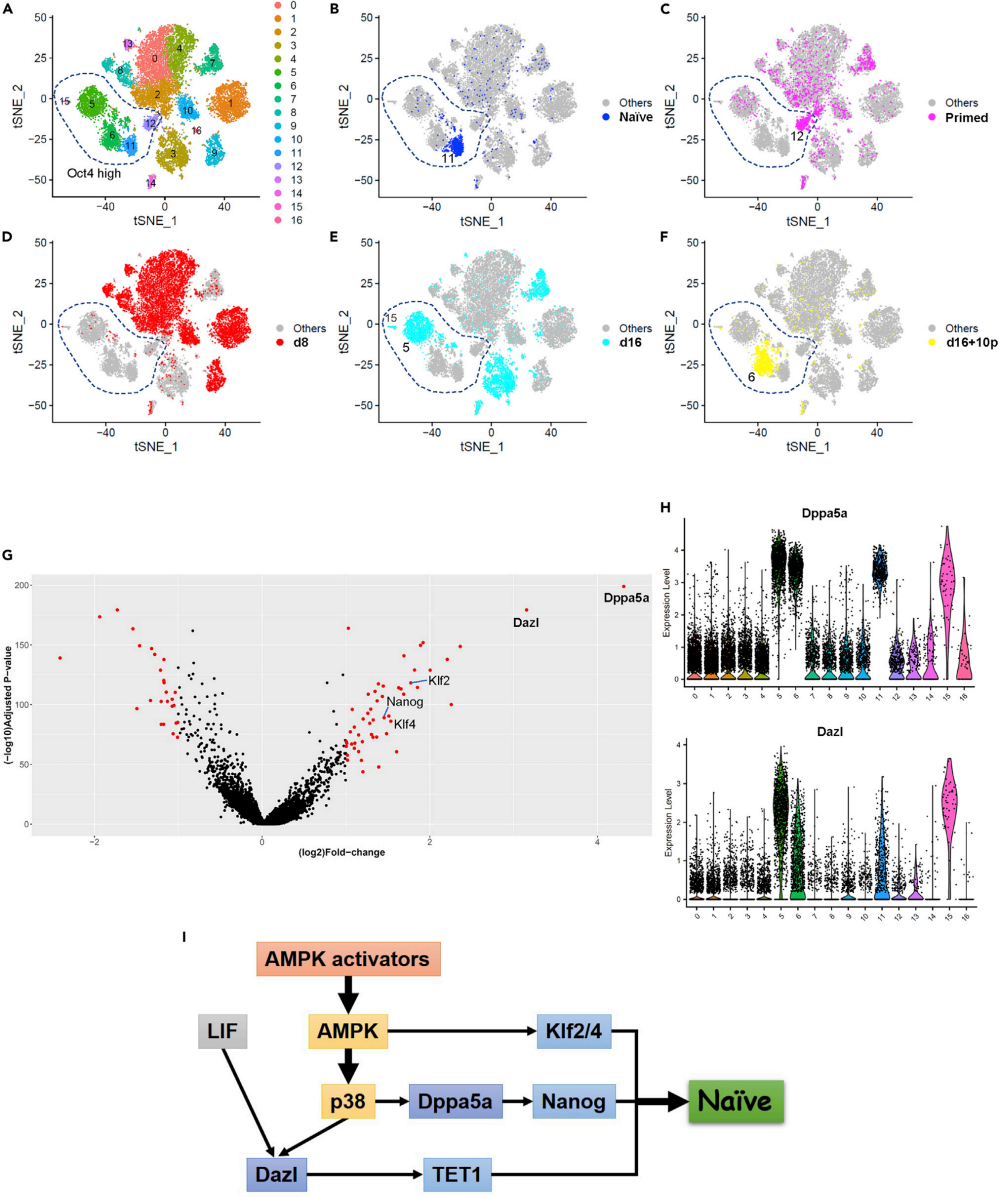
Single cell RNA-seq analysis during the reversion process (A–F) Two-dimensional t-SNE analysis for five cell populations during the naive reversion process. (A) t-SNE plot of all cell populations. A total of 15,628 of 17,720 cells after quality check were analyzed. Seventeen clusters (clusters 0–16) were obtained with the 2,000 most highly variable genes under the parameters of PCA dimension = 56, k neighbor = 50, and resolution = 0.8 by Seurat package version 4.0.1 in R version 4.0.3. Dotted line indicates clusters with high Oct4 expression (see Figure S6A). (B) Naive mESCs (Rex1GFP cells) (blue dots). (C) Primed mEpiSCs (Oct4GIP cells) (magenta dots). (D) Cells with AICAR + LIF treatment at d8 (red dots). (E) Cells with AICAR + LIF treatment at d16 (light blue dots). (F) AICAR + LIF-induced cells expanded in 2iL for 10 passages (yellow dots). (G) Volcano plot comparing gene expressions of cluster 5 and cluster 12. x axis: fold increase in cluster 5 compared with cluster 12 shown in log2 scale. y axis: adjusted p values shown in log10 scale. Genes with more than 2-fold differences are shown as red dots. (H) Violin plots for the Dppa5a and Dazl genes in each cluster. (I) Schematic summary of the putative mechanisms of naive reversion by AMPK activators. See also Figure S6.

Finally, we tried to elucidate the reversion process by AMPK activators using single-cell transcriptome analysis. We collected and analyzed the following 5 cell populations: naive mESCs carrying Rex1 promoter-driven GFPd2 gene (Rex1-GFP cells) (Bao et al., 2009; Wray et al., 2011), primed mEpiSCs (Oct4GIP cells), and cells with AICAR + LIF treatment at d8 (AICAR + LIF d8), at d16 (AICAR + LIF d16), and after expansion for 10 passages in 2iL condition (AICAR + LIF d16 + 10p). In total, 15,628 cells were analyzed. Cells were clustered into 17 populations by a t-distributed stochastic neighbor embedding (t-SNE) analysis (Figure 6A). Because clusters 5, 6, 11, 12, and 15 (inside the dotted line in Figure 6A) showed high and homogeneous Oct4 (gene name, Pou5f1) expression (Figure S6A), we considered these clusters as pluripotent stem cell populations including both naive and primed states. Cluster 11 represents naive mESCs (Rex1-GFP) (Figure 6B). Cluster 12 represents the main population of primed mEpiSCs (Oct4GIP) (Figure 6C), and other Oct4-negative cells in Figure 6C were considered MEF feeder cells. Eight days after treating primed mEpiSCs (Oct4GIP) with AICAR + LIF, most cells became negative for Oct4 (Figure 6D), suggesting they were the same as the cells that lost GFP expression around d8 after AICAR treatments (Figure S1A). At d16, when GFP^+^ cells re-appeared (Figure 1C), independent Oct4^+^ cluster (cluster 5 and 15) appeared (Figure 6E). After expanding these cells in 2iL condition for 10 passages, most moved to another independent Oct4^+^ cluster (cluster 6), the cluster nearest to naive mESCs (cluster 11) (Figure 6F). These results clearly demonstrated the AICAR-elicited reversion process from primed to naive cells, i.e.*,* after treatment with AICAR + LIF, GFP^+^ (Oct4^+^) primed cells (cluster 12) disappeared at d8, and then a small number re-appeared as a distinct population at d16 (cluster 5). After this intermediate reverting population (cluster 5) was cultured and expanded in 2iL condition, cells fulfilling various naive features (morphology, molecular markers, chimera formation, and germline transmission) were obtained (cluster 6). Cluster 11 (naive mESCs) and cluster 6 (AICAR-reverted cells) are very close but not identical, validating that these cells come from distinct cell lines with different genomic backgrounds.

Because cluster 5 at d16 is considered an intermediate naive cell population that represents the earliest GFP^+^ population after AICAR + LIF treatment, we compared the gene expression of cluster 5 and of mEpiSCs (cluster 12) to find specific genes upregulated after AICAR treatment. A volcano plot revealed that Dppa5a and Dazl were expressed higher (more than 16 and 8 times, respectively) in cluster 5 (Figure 6G). Violin plots for all clusters also showed that Dppa5a was highly expressed specifically in Oct4^+^ naive cell clusters (clusters 5, 6, 11, and 15) and Dazl was expressed highest in cluster 5 (Figure 6H). Critical transcription factors involved in the naive reversion, including Nanog, Klf2, and Klf4, were higher in cluster 5 than in cluster 12 (Figure 6G). Moreover, Nanog and Klf2 expressions were specifically elevated in clusters 5, 6, and 11. Klf4 expression was low in cluster 12 (Figure S6B). Thus, the single-cell analysis further elucidated the reversion process by AMPK activators, including the identification of an intermediate naive-state cell population in which Dppa5a and Dazl were highly upregulated. When AICAR and/or LIF was added to mEpiSCs, Dppa5a and Dazl mRNA expressions were increased, but the increase was blocked by p38i (Figure S6C). Taken together, Dppa5 and Dazl are critical candidate molecular factors downstream of AMPK and p38 in the naive reversion.

## Discussion

In this study, we demonstrated that AMPK activation successfully reverts primed mEpiSCs to naive state cells through the p38 pathway. The identification of a signaling pathway that can actively drive the reversion process provides valuable insights on the molecular machinery and relationship between the naive and primed pluripotency states. Unlike other reversion protocols (Table S1), our AMPK activator method uses no transgene but a single reagent. Thus, this study offers an important clue for narrowing down the reversion mechanism to a single key signaling pathway.

To our knowledge, this is the first report to identify the AMPK pathway in the reversion process from the primed to naive state. The AMPK pathway has been reported to promote reprogramming from somatic cells to pluripotent stem cells (Ma et al., 2015) and maintain naive pluripotency (Liu and Yamashita, 2019). We activated the AMPK pathway with three different AMPK activators: AICAR, A769662, and metformin. In most species, AMPK is an obligate heterotrimer that contains a catalytic subunit (α) and two regulatory subunits (β and γ) (Mihaylova and Shaw, 2012). AICAR mimics endogenous AMP as an AMP analog to activate the AMPK pathway by increasing intracellular AMP or the AMP/ATP ratio mainly through the α subunit (Mihaylova and Shaw, 2012). A769662 directly activates the β subunit to inhibit the dephosphorylation of AMPK (Göransson et al., 2007). Metformin leads to a drop of intracellular ATP levels by acting as a mild inhibitor of Complex I of the respiratory chain to activate AMPK (Mihaylova and Shaw, 2012). Despite their different activation mechanisms, all three compounds induced the appearance of naive-like cells from primed mEpiSCs via the AMPK pathway, indicating AMPK is a signaling molecule that can actively induce naive reversion. The efficiency of the AMPK activation and thus the reversion by these compounds are comparable but different (Figure S2A).

p38 is a downstream molecule of AMPK (Li et al., 2005). Activation of the p38 pathway has been reported to promote somatic cell reprogramming (Xu et al., 2013). We confirmed p38 activation in primed mEpiSCs with AMPK activators, blockade of the AMPK activator-elicited naive conversion by p38i, and partial reproduction of the naive reversion by p38 alone. All these results support the robustness of the reversion by the AMPK-p38 axis. Nevertheless, how the AMPK-p38 pathway is involved in naive and primed pluripotency molecular machinery requires further study. Transcription factor networks for pluripotency and epigenetic modifications are speculated as a central molecular mechanism regulating the pluripotency status (Bell et al., 2020). The relationship between these networks and the AMPK-p38 pathway should be further investigated to elucidate the AMPK-elicited naive reversion mechanism.

An AMPK activator (AICAR or A769662) alone or p38 activation alone, even in the absence of 2iL, achieved naive reversion, suggesting the potent driving force of the AMPK-p38 pathway to revert primed pluripotency to naive pluripotency. Nevertheless, the efficiency of the reversion was dependent on several culture conditions. First, during the first 16 days of AMPK activator treatment, MEFs were required for the maintenance of primed mEpiSCs. Without MEFs, most of cells disappeared until d10 (data not shown). Second, we used Basal medium, which includes 1% FBS and 10% knockout serum replacement (KSR). We have been using the Basal medium to maintain mESCs (Yamashita et al., 2000). The addition of 2iL to Basal medium resulted in the perfect maintenance condition for naive mESCs (Liu and Yamashita, 2019). However, when we tried Ndiff227 (N2B27 medium) instead for the reversion experiments, the cells showed differentiation morphology and loss of GFP expression, and the reversion was not observed even when treated with AMPK activators and LIF. Ndiff227 medium is a serum-free medium and used for the maintenance of naive pluripotent cells as well as neural and ectodermal cell differentiation (Ying et al., 2008). On the other hand, Basal medium contains serum components that may induce mesodermal lineage (Yamashita et al., 2000; Ying et al., 2008). Recently, BMP signal activation was reported to contribute to naive conversion (Stuart et al., 2019). Inferring from these results, in addition to AMPK activation, other factors, such as the presence of MEFs, serum, or BMPs, may be necessary to induce naive reversion efficiently.

Our single-cell RNA-seq analysis (Figure 6 and S6) provides further insights into the naive reversion caused by AMPK activation. First, the AMPK activators do not induce the reversion directly from the primed to naive state. Primed mEpiSCs first differentiated may be toward mesoderm or other germ layers before reverting to the naive state. Recently, a route from primed to naive pluripotency through a mesoderm state was reported (Stuart et al., 2019). The AMPK-elicited reversion may be using such a mesoderm route. Second, an intermediate naive state population, in which Dppa5 and Dazl are highly upregulated, was identified (Figures 6G and 6H). Dppa5 encodes structurally related proteins characterized by an atypical RNA-binding K Homology (KH) domain and has been reported to support human pluripotent stem cell self-renewal and reprogramming by stabilizing NANOG (Qian et al., 2016), a key transcription factor for naive reversion (Takashima et al., 2014). Dppa5 was also reported as a marker of human naive pluripotent stem cells (Kilens et al., 2018). Dazl, an RNA-binding protein that plays a key role in germ-cell development, enhances TET1-mediated cytosine hydroxymethylation in ESCs reverting to the naive state (Welling et al., 2015). Although AICAR or LIF alone did not increase the Dazl mRNA expression in mEpiSCs, AICAR + LIF did (Figure S6C), suggesting that LIF may promote the expression of Dazl with AMPK activators during the naive reversion. Taking all our observations together, we propose the putative naive reversion mechanism shown in Figure 6I.

In conclusion, our findings provide valuable insights on the molecular machinery regulating naive and primed pluripotency and thus contribute broadly to developmental biology and regenerative medicine including rejuvenation.

### Limitations of the study

Although we showed putative molecular links between AMPK, p38, Dppa5a, Dazl, and pluripotent transcription factors for naive reversion, their precise interactions at the molecular level were not clarified. Also, the roles of undefined factors from MEFs and serum are unknown. Finally, we used mouse cells for our experiments. This mechanism is yet to be confirmed in human cells.

## STAR★Methods

### Key resources table

**Table:** 

REAGENT or RESOURCE	SOURCE	IDENTIFIER
**Antibodies**		

Oct3/4	Santa Curz Biotechnology	Cat#sc-5279; RRID: AB_628051
Nanog	ReproCell	Cat# RCAB002P-F
Klf4	R&D Systems	Cat#AF3158; RRID: AB_2130245
Esrrb	Perseus Proteomics	Cat# PP-H6705-00; RRID: AB_1964232
Tfcp2l1	Invitrogen	Cat# PA5-34361; RRID: AB_2551713
APC rat anti-mouse CD31	BD Bioscience	Cat#551262; RRID: AB_398497
p-AMPK (T172)	Cell Signaling	Cat#2531S; RRID: AB_330330
AMPK	Cell Signaling	Cat#2532S; RRID: AB_330331
p-p38 MAPK (T180/Y182) (3D7)	Cell Signaling	Cat#9215S; RRID: AB_331762
p38 MAPK	Cell Signaling	Cat#9212S; RRID: AB_330713
β-actin	Sigma-Aldrich	Cat#A5441; RRID: AB_476744
Biotin-anti-mouse CD9	Biolegend	Cat#184803
Biotin-anti-mouse CD63	Biolegend	Cat#143918
TotalSeq™-A0951 PE streptavidin	Biolegend	Cat#405251
TotalSeq™-A0952 PE streptavidin	Biolegend	Cat#405253
TotalSeq™-A0953 PE streptavidin	Biolegend	Cat#405255
TotalSeq™-A0954 PE streptavidin	Biolegend	Cat#405257
TotalSeq™-A0955 PE streptavidin	Biolegend	Cat#405259

**Chemicals, peptides, and recombinant proteins**		

MLIF	Merck Millipore	Cat#ESG1107
PD0325901	Sigma-Aldrich	Cat#PZ0162-5MG; CAS: 391210-10-9
CHIR99021	Tocris	Cat#4423-10MG; CAS: 252917-06-9
FGF2	WAKO	Cat#060–05383; CAS RN: 106096-93-9
Activin A	R&D Systems	Cat#338-AC-01M
AICAR	WAKO	Cat#011–22533; CAS RN: 2627-69-2
A769662	ADooQ	Cat#A11071-10; CAS: 844499-71-4
Metformin	TCI Chemicals	Cat#M2009; CAS RN: 1115-70-4
SB203580	WAKO	Cat#199–16551; CAS RN: 152121-47-6
β-mercaptoethanol	GIBCO	Cat#21985023
DAPI	Invitrogen	Cat#D1306; CAS: 28,718-90-3
SYBR Green PCR master mix	Applied Biosystems	Cat#4367659
Puromycin	Nacalai	CAS RN: 58-58-2
Penicillin	Meji	Cat#4987-222-63,767-1
Streptomycin	Meji	Cat#4987-222-66,564-3
G418	Nacalai	CAS RN: 108321-42-2
Doxycycline	WAKO	CAS RN: 10,592-13-9
RNasin plus RNase inhibitor 2500U	Promega	Cat#N2611

**Critical commercial assays**		

RNeasy mini kit	QIAGEN	Cat#74104
RNase-free DNase set (50)	QIAGEN	Cat#79254
SuperScript first-strand synthesis SuperMix	Invitrogen	Cat#11752-050
SuperScript™ II reverse transcriptase 10,000 units	Invitrogen	Cat#18064014
Amaxa™ Mouse ES Cell Nucleofector™ Kit	Lonza	Cat#VPH-1001
Alkaline phosphatase staining kit II	Stemgent	Cat#00-0055
Agilent high sensitivity DNA kit	Agilent	Cat#5067-4626
HiSeq SR rapid cluster kit v2	Illumina	Cat#GD-402-4002
HiSeq rapid SBS kit v2	Illumina	Cat#FC-402-4022
TruSeq stranded mRNA library prep (48 samples)	Illumina	Cat#20020594
TruSeq RNA single indexes set a (12 indexes, 48 samples)	Illumina	Cat#20020492
TruSeq RNA single indexes set B (12 indexes, 48 samples)	Illumina	Cat#20020493

**Deposited data**		

RNA-seq	This study	GEO:GSE157087
Single-cell RNA-seq	This study	GEO:GSE175610

**Experimental models: cell line**		

Oct4GIP mEpiSC (XX)	(Guo et al., 2009)	N/A
129/Ba1 mEpiSC (XY)	(Sugimoto et al., 2015)	N/A
129/MSM mEpiSC (XY)	(Yagi et al., 2019)	N/A
Rex1-GFP mESC (XX)	(Kalkan et al., 2017)	N/A

**Oligonucleotides**		

Primers for qPCR, see Table S2	This study	N/A

**Recombinant DNA**		

ca-p38 cDNA	Gift from X.Xu	N/A
PB-TetO-IRES-mcherry-Ef1a-rtTA-IRES-neo	(Tanaka et al., 2013)	N/A
pENTR-GOI	(Tanaka et al., 2013)	N/A

**Software and algorithms**		

Image J	(Schneider et al., 2012)	https://imagej.nih.gov/ij/

### Resource availability

#### Lead contact

Further information and requests for resources and reagents should be directed to and will be fulfilled by the lead contact, Professor Jun K. Yamashita (juny@cira.kyoto-u.ac.jp).

#### Materials availability

Further information and requests for resources and reagents should be directed to and will be fulfilled by the lead contact.

#### Data and code availability

The accession number for RNA-seq data reported in this paper is GEO: GSE157087.

The accession number for single cell RNA-seq data reported in this paper is GEO: GSE175610.

### Method details

#### Cell lines and culture

We used three different mouse epiblast stem cell lines: Oct4GIP (Ying and Smith, 2003; Guo et al., 2009), 129/MSM (Yagi et al., 2019), 129/Ba1 (Sugimoto et al., 2015) and one naïve embryonic stem cell line Rex1-GFP (Masui et al., 2008; Kalkan et al., 2017) as positive control in our study. The mouse epiblast stem cell (mEpiSC) line Oct4GIP was cultured on fibronectin (Life technologies)-coated (37°C, 1 hr) plates (10 μg/mL/cm^2^) as described previously (Ying and Smith, 2003; Guo et al., 2009). In brief, cells were expanded in Ndiff 227 (TaKaRa Bio) medium supplemented with FGF2 (12 ng/mL, WAKO) and Activin A (20 ng/mL, R&D) and replated every 3–4 days by dissociation with Accumax (Innovative Cell Technologies). The medium was changed every 2 days. 129/MSM was a gift from Dr. Masaki Yagi (Yagi et al., 2019). In brief, male MSM/Ms mice were mated with female 129X1/SvJ mice. Noon of the day when the plug was observed was designated as embryonic day (E) 0.5. The day before isolating the embryos, mitomycin-C (Kyowa Hakko Kirin Co., Ltd.)-treated MEFs (feeders) were harvested on 0.2% gelatin-coated 24-well culture plates (True Line). At E6.5, the uterus was removed by cutting across the cervix and the two uterotubal junctions, and the embryos were placed in HEPES (GIBCO). The muscle layer was then removed, and the deciduum was dissected using needles. Epiblasts were divided from the extra-embryonic regions and transferred into prepared 24-well culture plates to derive EpiSCs. After outgrowth of the epiblasts, the cells were passaged on feeders in 24-well culture plates (p1), 6-well culture plates (p2), and 6-cm culture dishes (p3). 129/MSM were cultured on MEF feeders in DMEM/F-12 medium (GIBCO) supplemented with 20% Knockout Serum Replacement (KSR) (GIBCO), 0.1 mM MEM non-essential amino acids (NEAA) (GIBCO), 0.1 mM 2-mercaptoethanol (GIBCO), and penicillin/streptomycin, and supplemented with FGF2 (12 ng/mL) and Activin A (20 ng/mL). The medium was changed every 2 days. 129/Ba1 mEpiSCs were obtained as described previously (Sugimoto et al., 2015). In brief, cells were expanded on MEF feeder in DMEM/F-12 supplemented with 15% KSR, 0.1 mM NEAA, 0.1 mM 2-mercaptoethanol, penicillin/streptomycin, FGF2 (12 ng/mL) and Activin A (20 ng/mL). The medium was changed every 2 days. naive mouse embryonic stem cells (mESCs) and naïve-like cells after the reversion of mEpiSCs were cultured on 0.1% gelatin-coated dishes as described previously (Yamashita et al., 2000; Ying et al., 2008) in Basal medium (GMEM (GIBCO) supplemented with 10% KSR (GIBCO), 1% fetal bovine serum (SAFC Biosciences), 0.1 mM NEAA, 1 mM sodium pyruvate (SIGMA), 0.1 mM 2-mercaptoethanol, and penicillin/streptomycin)) or in Ndiff 227 medium. Both media were supplemented with 2iL: 1000 U/mL Lif (Millipore) and two small molecule inhibitors (1 μM PD0325901 (SIGMA) and 3 μM CHIR99021 (Tocris). Cultures were passaged every 3–4 days by dissociation with Accumax. The medium was changed every 2 days.

#### mEpiSC reversion by AMPK activators

mEpiSCs dissociated with Accumax were plated on MEF feeders in Ndiff 227 medium supplement with FGF2 (12 ng/mL) and Activin A (20 ng/mL) for 1 day (d-1-d0). The medium was changed to Basal medium and the various combinations of reagents shown in Figure 1A and Table 1 at d0 for 16 days (d0-d16). The AMPK activator used was AICAR (1 mM, dilution in D_2_W, WAKO), A769662 (50 μM, dilution in DMSO, ADooQ), or metformin (1 mM, dilution in D_2_W, TCI). The medium was changed every 2 days. Because AICAR has some growth inhibitory effects (Chae et al., 2012) and the cell number decreased after long-time culturing with AICAR, in the first 6 days (d0-d6), the concentration of AICAR was 0.5 mM. After 16 days, the cells were dissociated with Accumax for further analysis or expansion on gelatin-coated 6-well plates in Basal medium or Ndiff227 medium with 2iL. The medium was replaced every other day and passaged every 4–7 days for at least 3 passages until stable colonies developed. A p38 inhibitor, SB203580 (10 μM, WAKO) was added 1–2 hr before AMPK activator treatment.

#### FACS analysis

Cells were washed twice with PBS and harvested using Accumax, then stained with allophycocyanin (APC)-conjugated anti-CD31 (PECAM1) MoAb (BD) and DAPI (Invitrogen). Flow cytometry analysis was performed on a FACSAriaII Cell Sorter (BD). All FACS experiments were repeated at least three times.

#### Immunostaining and alkaline phosphatase staining

Immunostaining was carried out as described previously (Yamashita et al., 2000). In brief, cells were fixed in 4% paraformaldehyde for 15–20 min, washed 3 times with PBS and blocked with 2% skim milk (BD) for 30 min. The cells were then incubated with primary antibodies overnight at 4°C. The next day, the cells were rinsed 3 times with PBST (PBS+0.02%Tween 20, Nakalai tesque) and treated for 1 hr at room temperature with secondary antibodies, anti-mouse, rabbit, or goat IgG antibodies conjugated with Alexa 488 or Alexa 546 (Invitrogen) diluted 1:500. Nuclei were stained with DAPI. The following antibodies were use at the indicated dilutions: anti-Oct3/4 (Santa Cruz, sc-5279, 1:200), anti-Nanog (ReproCell, RCAB002P-F, 1:300), anti-KLF4 (R&D, AF3158, 1:500), anti-ESRRB (Perseus Proteomics, PP-H6705-00 1:500), anti-TFCP2l1 (Invitrogen, PA5-34361, 1:400), anti-TFE3 (Sigma, HPA023881, 1:300), anti-FOXA2 (Merck Millipore,07–633, 1:500), anti-Brachyury (R&D, AF2085, 1:500), and anti-Nestin (StemCell Technologies, 01,418, 1:500). For alkaline phosphatase staining, the AP staining kit II (Stemgent) was used. According to the manufacturer's instructions, cells were fixed with 4% paraformaldehyde for 5 min, washed once in PBST, stained with Solution A + B + C for 10 min, and washed three times in PBS. For single cell colony formation, 500 cells were plated in 12-well plates and cultured for 5 days.

#### RNA isolation and quantitative PCR

Total RNA was isolated using the RNeasy Mini Kit (QIAGEN), and cDNA was reversed-transcribed from 1 μg RNA using SuperScript III (Invitrogen). Quantitative PCR analysis was performed in duplicates using 1/50 of the reverse transcription reaction in StepOnePlus (Applied Biosystems) with SYBR Green Master Mix (Applied Biosystems). All qPCR reactions were performed in triplicate from at least 3 independent experiments. An endogenous control GAPDH was used to normalize the gene expressions. All results are presented as mean ± SD. The primers used are listed in Table S2.

#### Western blots

Cells were lysed in Sample buffer solution with 2-ME (Nacalai Tesque). Cell lysates were run on e-*PAGEL* Gel (ATTO) and electrophoretically transferred onto nitrocellulose membranes. The cells were then blocked for 30 min in Blocking One (Nacalai Tesque) and incubated overnight at 4°C with primary antibodies for the following targets: phosphorylated-AMPK (Thr172, Cell Signaling, 2531S, 1:1000), AMPK (Cell Signaling, 2532S, 1:1000), phosphorylated-p38 (Thr180/Tyr182, Cell Signaling, 9215S, 1:1000), p38 (Cell Signaling, 9212S, 1:1000), and β-actin (Sigma, A5441, 1:5000). The secondary antibodies were anti-mouse (Invitrogen, 62–6520, 1:50,000) and rabbit IgG (Cell Signaling, 7074S,1:3000-1:1000) antibodies conjugated with Horseradish peroxidase (HRP). After 2 hr incubation at room temperature, immobilon western chemiluminescent substrate (Millipore) was used for visualization and ImageQuant LAS4000 was used for detection. All primary and secondary antibodies were diluted using a Can Get Signal Immunoreaction Enhancer Solution Kit (Toyobo). Blot results were quantified by ImageJ.

#### Generation of constitutively active p38 cell line

p38 cDNA containing D176A and F327S mutations (Xu et al., 2013; Diskin et al., 2004) was inserted into a piggyBac (PB) vector carrying rtTA expression coupled to mCherry (Tanaka et al., 2013). Using the Nucleofector transfection system (Amaxa), we introduced Tet-On CA-p38 with mCherry gene into the Oct4GIP cell line. Cells were plated on a 6-cm dish maintained with FGF2 (12 ng/mL) and Activin A (20 ng/mL) for 3 days and then G418 (400 μg/mL) was applied for the selection. The induction of CA-p38 was checked with the expression of mCherry. After 24 hr of doxycycline (dox) treatment from d8 after the transfection, mCherry^+^ cells were sorted and maintained again in Ndiff 227 medium with FGF2, Activin A, and G418 in the absence of dox. Western blot analysis confirmed the p38 pathway was activated in the dox + condition (Figure S5F).

#### Chimera blastocyst injection and germline transmission

All animal experimental protocols were approved by the Animal Experimentation Committee, Kyoto University. All animal experiments were performed according to the ‘*Guidelines for Animal Experiments of Kyoto University*’, which conforms to Japanese law and ‘*the Guide for the Care and Use of Laboratory Animals*’. We used a male cell line, 129/Ba1, generated from 129/Sv×C57BL/6N mice (Sugimoto et al., 2015). Cells were pre-cultured on MEF feeder with FGF2 and Activin A for 1 day (d(-1)-d0) and then in Basal medium with AICAR or AICAR + LIF (d0-d16). After d16, the cells were re-cultured in 2iL condition for several passages and used in the blastocyst injection. Host blastocysts were isolated from Slc:ICR female mice, and no more than 12 reverted cells were injected into the blastocoel cavity using a Piezo micromanipulator (Primetech, Japan). Injected blastocysts were transferred to 2.5 days postcoitum pseudopregnant females. Chimeric mice were determined by their coat color. When chimeric mice became sexually mature 8 weeks post coitum, they were mated with wild type ICR mice to confirm germline transmission. In the next generation, if agouti coated mice were born, the germline transmission was considered successful.

#### Imaging analysis

An inverted microscope (Olympus IX71) equipped with U-RFL-T (Olympus) was used for bright field and live-cell fluorescence imaging. An LSM700 inverted confocal microscope (Zeiss) equipped with 405 nm, 488 nm, 556 nm and 635 nm lasers using ×10 or ×20 objective was used for immunostaining in the figures.

#### RNA sequencing

The sequencing libraries were constructed using the TruSeq Stranded mRNA Library Prep (Illumina, Inc.), and 500 ng of total RNA were sequenced using the 79 cycle single read mode of HiSeq2500. All sequenced reads that passed quality filters were extracted to FASTQ format and demultiplexed to individual cells by barcodes using BCL2FASTQ Conversion Software v2.20.0.422. FASTQ converted reads were then mapped to Ensembl GRCm38 release 100 reference cDNA and ncRNA sequences using Bowtie2 v2.2.5 with the very-sensitive-local option. Among a total of 68,196,501 reads, 62,621,611 (91.8%) were successfully mapped and quantified as genes with a threshold of MAPQ score ≥1. A total of 34,489 genes (21,500 on average) were detected for 22 samples. The following analyses were performed using R ver. 3.6.3 after non-categorized limma voom normalization (Law et al., 2014). A total of 22 samples from naive ESCs, primed EpiSCs, reverted cells (d16+2p, 3p, 10p) and cells in the reversion process (d8, 16) were analyzed. A PCA of the 22 samples for 2036 pluripotent cell fate (PCF) genes, except BC052688 (Fidalgo et al., 2016), and all 34,489 genes are shown in Figures 3A and S4, respectively. The heatmap (Figure 3B) was analyzed for a selected gene set of pluripotency regulators and lineage markers (Takashima et al., 2014) as depicted.

#### Single-cell RNA sequencing

The following five samples were collected: 500 naive mESCs (Rex1GFP), 500 primed mEpiSCs (Oct4GIP), 9000 AICAR + LIF d8 cells, 9000 AICAR + LIF d16 cells, and 1000 AICAR + LIF d16 + 10p cells as determined using FlowCount beads (Beckman Coulter) and a combination of Biotin-CD9 and Biotin-CD63 (Biolegend) conjugated with TotalSeq-A095(1–5) PE Streptavidin. A total of 20,000 cells were collected into one tube, and immediately after a cDNA trap was performed using BD Rhapsody, cDNA was synthesized, and free-primer digestion was done using ExoI. TAS-Seq was then performed. Sequencing was entrusted to ImmunoGeneTeqs (Chen et al., 2020). Adapter trimming of the sequencing data was performed using cutadapt 2.10. Filtered reads were chunked to 16 parts for parallel processing using Seqkit 0.9.0. Filtered cell barcode reads were annotated by Python scripts provided by BD with minor modification so that the reads were compatible with Python3.7. The associated cDNA reads were mapped to Ensembl cDNA and ncRNA (build GRCm38.p6, release-100) using Bowtie2-2.3.4.1 and the following parameters: -N 1 --very-sensitive-local --seed 656565 –reorder. Associated hashtag reads were mapped to known barcode sequences + BAAAAA using Bowtie2-2.4.2 and the following parameters:-D 50 -R 20 -N 0 -L 8 -i S,1,0.75 --norc --seed 656565 --reorder --trim-to 3:21 --score-min L,-9,0 --mp 3,3 --np 3 --rdg 3,3. Then, the cell barcode information of each read was added to the bowtie2-mapped BAM files, and read counts of the same gene symbols of each cell barcode were counted. The inflection point of the knee-plot of the cell barcode/read number was detected using the DropletUtils package in R 3.5.3, and cell barcodes below the inflection threshold were filtered out. Each hashtag read was normalized by the total read counts into the smallest total read counts linearly, and each cell was annotated to each hashtag based on the fold-change between the first- and second-most highly counted hashtags. Then, the cells were ordered ascending by the fold-change, and the top 4.13% cells were filtered out as doublet cells (the probability of the doublet was estimated by Poisson's distribution calculated by the load cell number and total number of Rhapsody wells). Preparation of the sequence data was performed in CiRA, Kyoto University and a contract analysis with ImmnoGeneTeqs, Inc., Japan.

In Figures 6, 15,628 of 17,720 cells after quality check were normalized and analyzed by the Seurat package version 4.0.1 in R version 4.0.3. Seventeen clusters (clusters 0–16) were obtained with the 2000 most highly variable genes under the parameters of PCA dimension = 56, k neighbor = 50, and resolution = 0.8, and two-dimensional t-SNE and violin plots were drawn by the package. Volcano plots between clusters 5 and 12 for all 33,766 genes were also drawn using ggplot2 package in R.

### Quantification and statistical analysis

For experiments comparing differences among different groups, we used one-way analysis of ANOVA by Tukey's Multiple Comparison Test. Differences were considered significant for p values <0.05. All experiments were performed at least 3 times independently. ImageJ was used to quantify the Western blot results. Error bars indicate standard deviations.

## Supplemental information

Document S1. Figures S1–S6

Table S1. Components of reported protocols for naive reversion (mouse cells), related to discussion

Table S2. Primer sequences for qPCR, related to STAR Methods, Figure 2, S3, and S6
