# Supplementary material for: AMPK activation reverts mouse epiblast stem cells to naive state
Source: iScience. 2021 Jun 25;24(7):102783. doi: 10.1016/j.isci.2021.102783 (PMC8283141; doi:10.1016/j.isci.2021.102783)
Supplement: Document S1. Figures S1–S6 [file mmc1.pdf]

**iScience, Volume *24***

## Supplemental information

# AMPK activation reverts mouse epiblast stem cells to naive state

**Yajing Liu, Junko Yamane, Akito Tanaka, Wataru Fujibuchi, and Jun K. Yamashita**

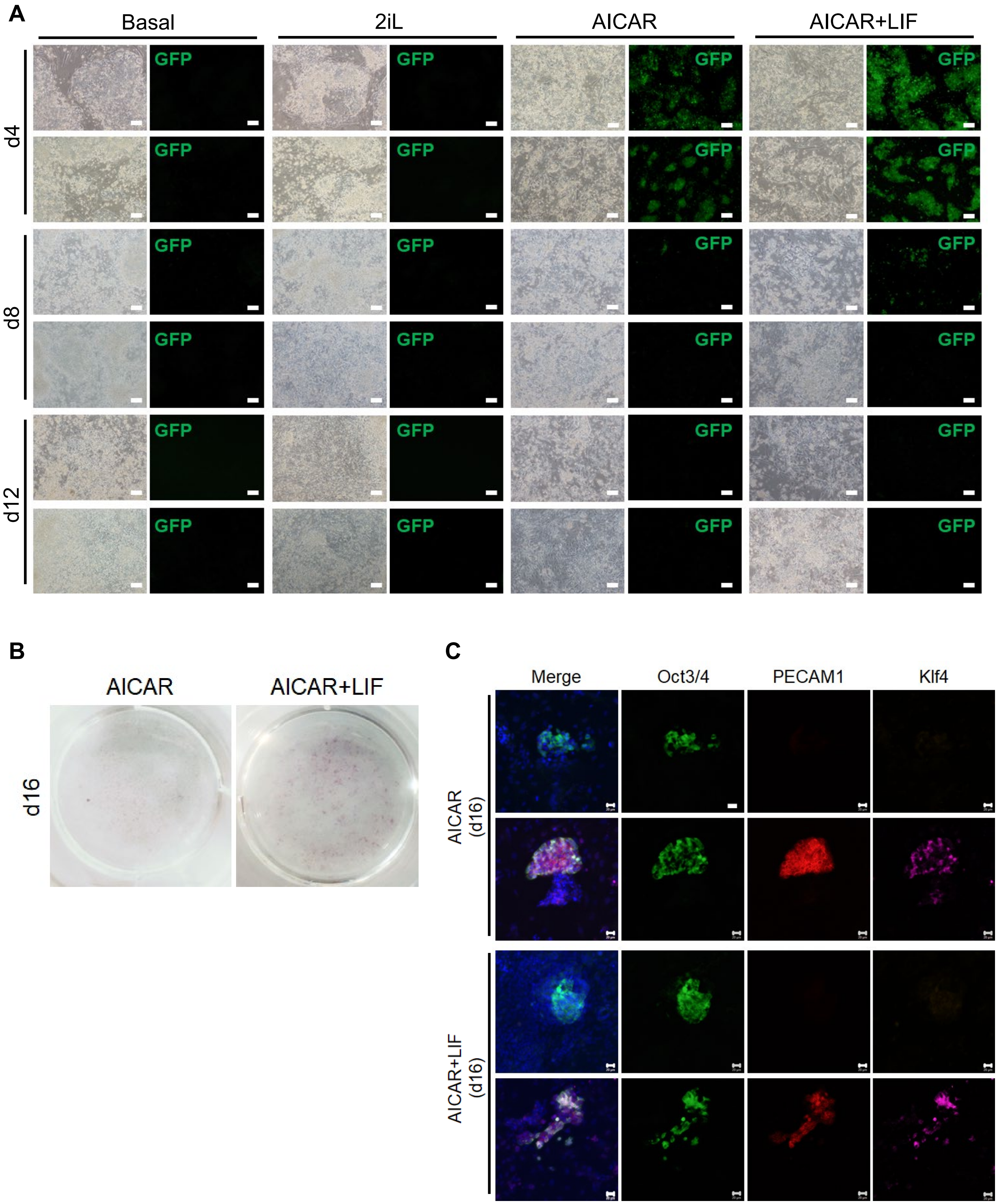
A
Basal
2iL
AICAR
AICAR+LIF
d4
d8
d12
GFP
B
AICAR
AICAR+LIF
d16
C
Merge
Oct3/4
PECAM1
Klf4
AICAR (d16)
AICAR+LIF (d16)

**Figure S1. AICAR Induces the Reversion of Primed mEpiSCs to Naïve-Like Cells, Related to Figure 1**

(A) Cell morphology and GFP expression of mEpiSCs (Oct4GIP) after treatment with Basal medium alone, 2i/L, AICAR, or AICAR+LIF day(d) 4, 8 and 12. Two pairs of phase contrast and Oct4-GFP images are shown for each condition. Scale bars, 200 μm.

(B) AP staining of Oct4GIP reverted by AICAR or AICAR+LIF after 16 days (d16). (Cells were treated with AICAR or AICAR+LIF after 16 days, and then AP staining was performed).

(C) Immunofluorescence staining for pluripotent and naïve markers Oct4 (green), PECAM1 (red) and Klf4 (purple) in cells 16 days of treatment with AICAR or AICAR+LIF. DAPI (blue), nuclear staining. Scale bars, 20 μm.

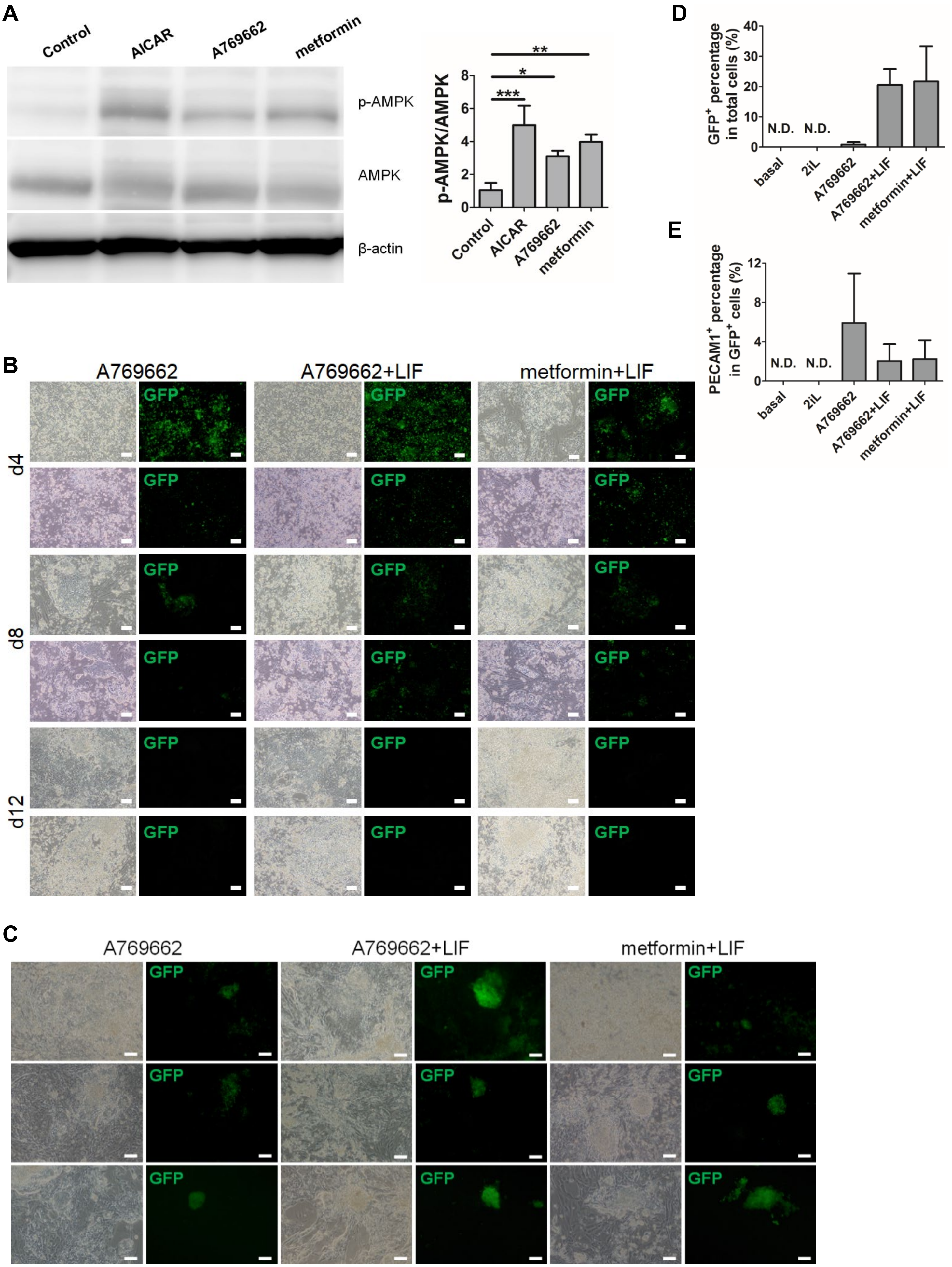
A
Control
AICAR
A769662
metformin
p-AMPK
AMPK
β-actin
p-AMPK/AMPK
***
*
**
D
GFP+ percentage in total cells (%)
N.D.
N.D.
basal
2iL
A769662
A769662+LIF
metformin+LIF
E
PECAM1+ percentage in GFP+ cells (%)
N.D.
N.D.
basal
2iL
A769662
A769662+LIF
metformin+LIF
B
A769662
A769662+LIF
metformin+LIF
d4
d8
d12
GFP
C
A769662
A769662+LIF
metformin+LIF
GFP

**Figure S2. A769662 and Metformin Induce the Reversion of primed mEpiSCs to Naïve-Like Cells, Related to Figure 1**

(A) Western blots for AMPK activation by three AMPK activators, AICAR (1 mM), A769662 (50 μM) and metformin (1mM) for primed mEpiSCs (Oct4GIP). p-AMPK, phosphorylated AMPK (activated); AMPK, total AMPK. Left panels: representative blots. Right graph: Quantitative evaluation of the p-AMPK/AMPK density ratio. Control samples were set to 1. (mean±SD, n=3, *$p$<0.05, **$p$<0.01, ***$p$<0.001, one-way ANOVA followed by Tukey's multiple comparison test.)

(B) Cell morphology and Oct4-GFP expression of Oct4GIP after treatment with A769662, A769662+LIF, or metformin+LIF on day(d) 4, 8 and 12. Two pairs of phase contrast and Oct4-GFP images are shown for each condition. Scale bars, 200 μm.

(C) Cell morphology and Oct4-GFP expression of Oct4GIP after treatment with A769662, A769662+LIF, or metformin+LIF for 16 days. Three pairs of phase contrast and Oct4-GFP images are shown for each condition. Scale bars, 200 μm.

(D) and (E) Quantitative evaluation of Oct4-GFP-positive cells in total cells and PECAM1-positive cells in Oct4-GFP-positive cells obtained by FACS analysis (n=4; N.D., not detected).

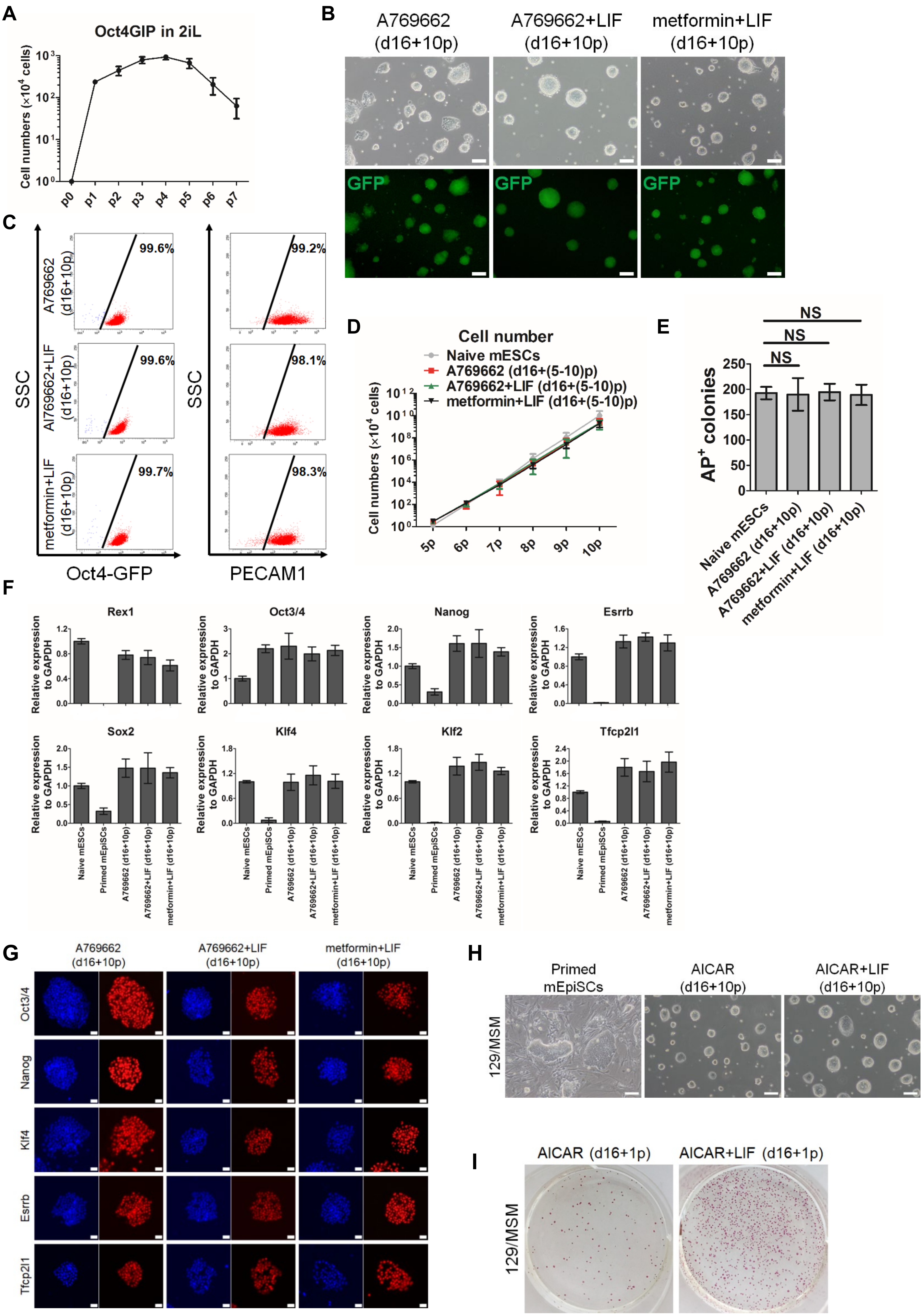

A
Oct4GIP in 2iL
Cell numbers (×10^4 cells)
p0 p1 p2 p3 p4 p5 p6 p7
B
A769662 (d16+10p)
A769662+LIF (d16+10p)
metformin+LIF (d16+10p)
GFP
C
A769662 (d16+10p)
AI769662+LIF (d16+10p)
metformin+LIF (d16+10p)
99.6%
99.6%
99.7%
99.2%
98.1%
98.3%
SSC
Oct4-GFP
PECAM1
D
Cell number
Naive mESCs
A769662 (d16+(5-10)p)
A769662+LIF (d16+(5-10)p)
metformin+LIF (d16+(5-10)p)
Cell numbers (×10^4 cells)
5p 6p 7p 8p 9p 10p
E
NS
NS
NS
AP+ colonies
Naive mESCs
A769662 (d16+10p)
A769662+LIF (d16+10p)
metformin+LIF (d16+10p)
F
Rex1
Oct3/4
Nanog
Esrrb
Sox2
Klf4
Klf2
Tfcp2l1
Relative expression to GAPDH
Naive mESCs
Primed mEpiSCs
A769662 (d16+10p)
A769662+LIF (d16+10p)
metformin+LIF (d16+10p)
G
A769662 (d16+10p)
A769662+LIF (d16+10p)
metformin+LIF (d16+10p)
Oct3/4
Nanog
Klf4
Esrrb
Tfcp2l1
H
Primed mEpiSCs
AICAR (d16+10p)
AICAR+LIF (d16+10p)
129/MSM
I
AICAR (d16+1p)
AICAR+LIF (d16+1p)
129/MSM

**Figure S3. AMPK-Induced Reverted Cells Show Naïve Pluripotency Features, Related to Figure 2**

(A) Cell growth of Oct4GIP in 2iL. Plates were first coated with MEF, then the cells were cultured in Ndiff 227+2iL condition for several passages (mean ± SD; n=3).
(B) Cell morphology and Oct4-GFP expression in cells reverted with A769662, A769662+LIF, or metformin+LIF after expansion in 2iL condition (d16+10p). Scale bars, 200 μm.
(C) FACS analysis for Oct4-GFP and PECAM1 in reverted cells maintained by A769662, A769662+LIF or metformin+LIF (d16+10p). Percentages are Oct4-GFP-positive or PECAM1-positive cells in total cells.
(D) Growth of reverted cells. Numbers of reverted cells during passages 5 to 10 compared with naïve mESCs in 2iL (mean ± SD; n=4).

(E) $AP^+$ colony formation assay. Five hundred reverted cells (d16+10p) or naïve mESCs were plated in 2iL condition. After 5 days of culture, AP-stained colonies were counted (mean ± SD; n=4, NS: not significant).
(F) Naïve and pluripotent gene mRNA expressions (qPCR) in naïve mESCs, primed mEpiSCs (Oct4GIP) and cells reverted by A769662, A769662+LIF or metformin+LIF (d16+10p). Naïve ESCs: Rex1-GFP cells maintained in 2iL. Expression levels are normalized to GAPDH. Data are represented as mean ± SD (n=4; with technical triplicates). The results of naïve mESCs were set to 1.

(G) Immunofluorescence staining for naïve and pluripotent markers (red) in cells reverted by A769662 A769662+LIF or metformin+LIF. (d16+10p). DAPI (blue), nuclear staining. Scale bars, 20 μm.

(H) Cell morphology of primed mEpiSC line 129/MSM and 129/MSM cells reverted by AICAR or AICAR+LIF (d16+10p). Scale bars, 200 μm.

(I) AP staining of reverted 129/MSM cells (d16+1p) cultured in 2i/L for 7 days. (Cells were harvested after 16 days reversion (d16), then cultured in 2i/L for 7 days, and finally AP staining was performed.)

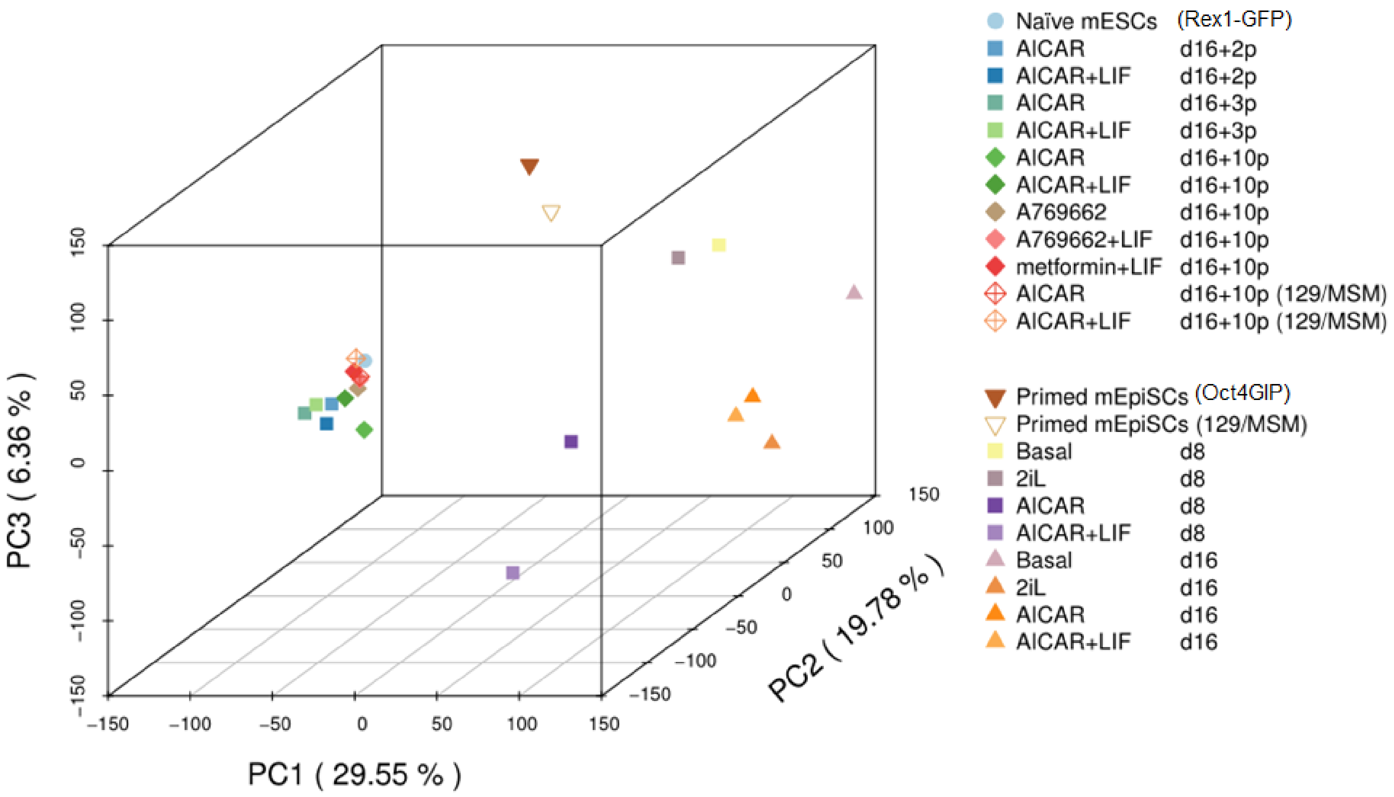


**Figure S4. RNA-seq Analysis During the Reversion Process, Related to Figure 3**

PCA of all gene signatures (34489 genes) in different cell types. Naïve mESCs: Rex1-GFP cells; Primed mEpiSCs: Oct4GIP cells; and Oct4GIP reverted by AMPK activators and cultured in 2iL condition after different passages: AICAR, AICAR+LIF (d16+2p, d16+3p, d16+10p), A769662, A769662+LIF, or metformin+LIF (d16+10p). Oct4GIP or 129/MSM cells treated in Basal medium, 2iL, AICAR, or AICAR+LIF for several days (d) and several passages (p). Reverted cells shared similar gene signatures with naïve mESCs.

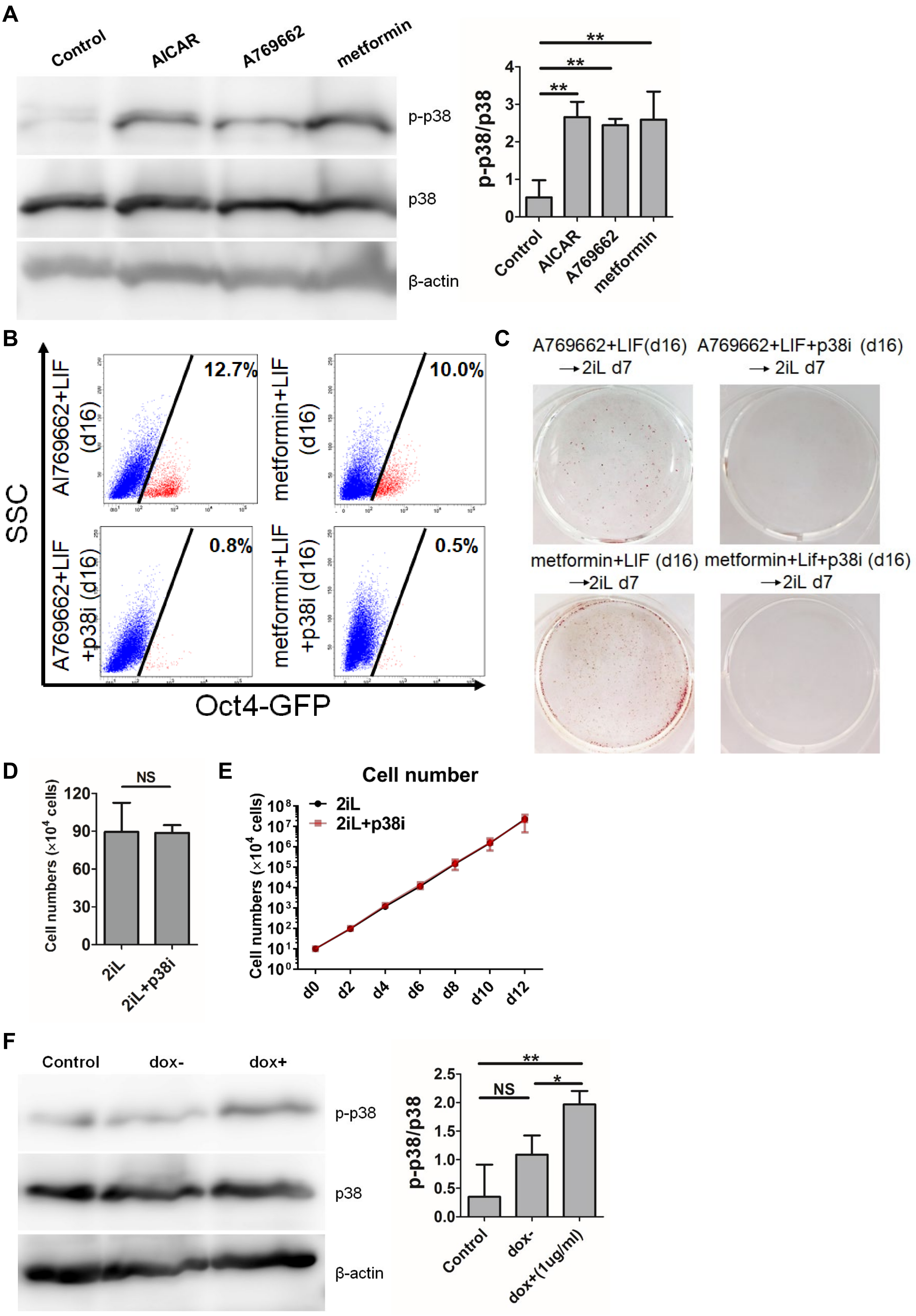
A
Control
AICAR
A769662
metformin
p-p38
p38
β-actin
p-p38/p38
**
**
**
Control
AICAR
A769662
metformin
B
SSC
Oct4-GFP
A769662+LIF (d16)
12.7%
metformin+LIF (d16)
10.0%
A769662+LIF +p38i (d16)
0.8%
metformin+LIF +p38i (d16)
0.5%
C
A769662+LIF(d16) →2iL d7
A769662+LIF+p38i (d16) → 2iL d7
metformin+LIF (d16) →2iL d7
metformin+Lif+p38i (d16) →2iL d7
D
NS
Cell numbers (×10⁴ cells)
2iL
2iL+p38i
E
Cell number
2iL
2iL+p38i
Cell numbers (×10⁴ cells)
d0
d2
d4
d6
d8
d10
d12
F
Control
dox-
dox+
p-p38
p38
β-actin
p-p38/p38
**
NS
*
Control
dox-
dox+(1ug/ml)

**Figure S5. Involvement of p38 in the AMPK Pathway, Related to Figure 5**

(A) p38 activation with AMPK activators. Western blots for p38 activation in primed mEpiSCs (Oct4GIP) cultured with AICAR (1 mM), A769662 (50 μM) or metformin (1 mM). p-p38, phosphorylated p38 (Thr180/Tyr182); p38, total p38. Left panels: representative blots. Right graph: Quantitative evaluation of the p-p38/p38 density ratio. The control samples were set to 1 (mean±SD, n=3, *$p<0.05$, **$p<0.01$, one-way ANOVA followed by Tukey's multiple comparison test).

(B) FACS analysis of bulk cells cultured in 2iL for 7 days after reversion by A769662+LIF, A769662+LIF+p38i, metformin+LIF, or metformin+LIF+p38i. Red represents the Oct4-GFP positive fraction.

(C) AP staining of bulk cells cultured in 2iL for 7 days after reversion by A769662+LIF, A769662+LIF+p38i, metformin+LIF, or metformin+LIF+p38i.

(D) Number of Oct4GIP AICAR+LIF (d16) cells cultured in 2iL or 2iL+p38i. $10\times10^4$ Oct4GIP AICAR+LIF (d16) cells were maintained in 2iL or 2iL+p38i. After 5 days, the cells were harvested, and the cell numbers were counted (mean ± SD; n=3; NS: not significant).

(E) Growth of naïve mESCs (Rex1GFP) in 2iL or 2iL+p38i. $10\times10^4$ cells were cultured in 2iL or 2iL+p38i for 12 days and passaged every 2 days (mean ± SD; n=5).

(F) Activation of the p38 pathway by doxycycline-inducible (dox-+) constitutively active p38 (CA-p38). Western blots for p-p38, p38, and β-actin after treatment with or without dox (1 μg/mL) treatment. Control: original Oct4GIP cells. Left panels: representative blots. Right graph: Quantitative evaluation of the p-p38/p38 density ratio. The dox- sample was set to 1 (mean± SD, n=3, *$p<0.05$, **$p<0.01$, NS: not significant).

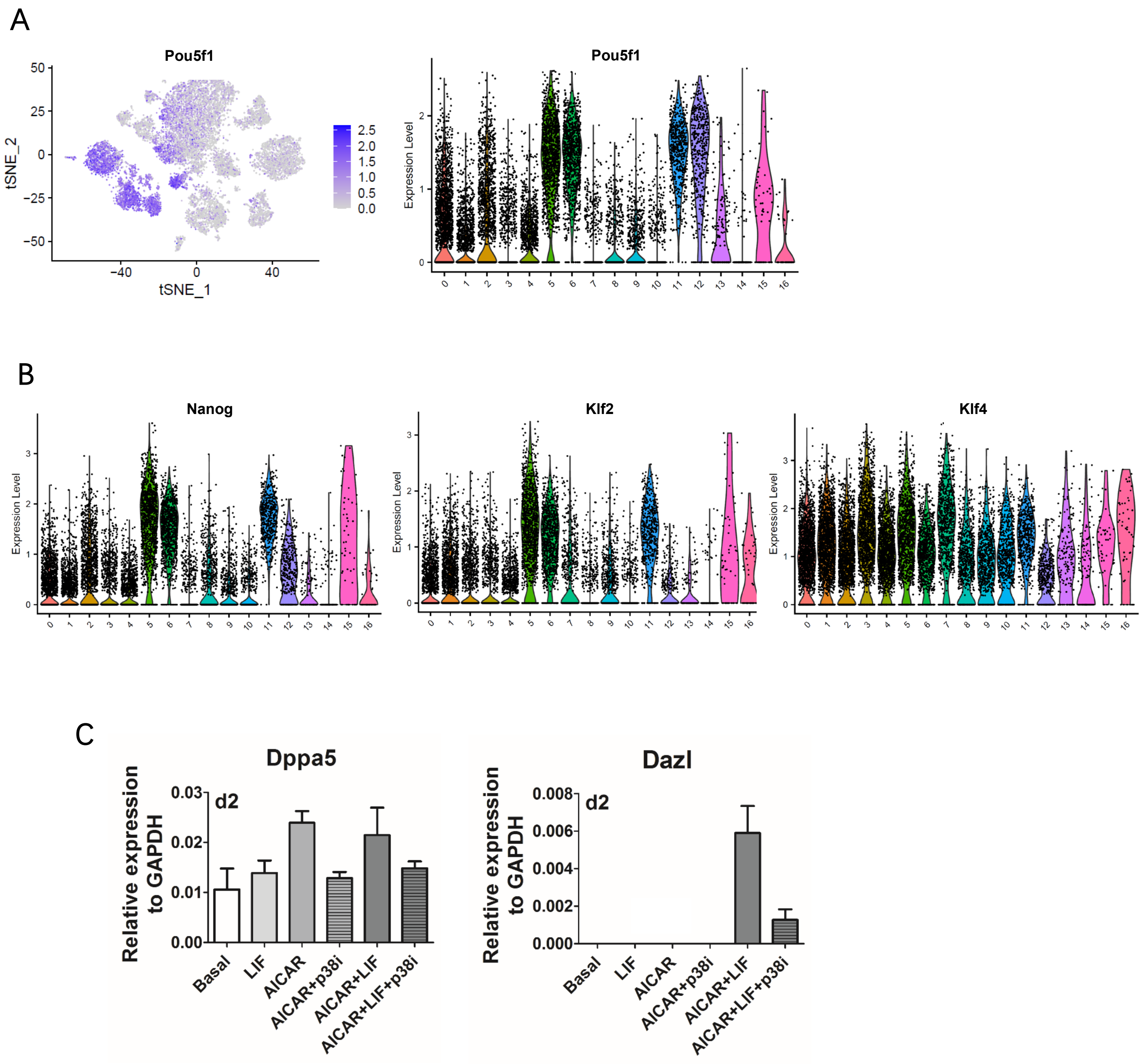

A
Pou5f1
tSNE_2
tSNE_1
Pou5f1
Expression Level
B
Nanog
Klf2
Klf4
Expression Level
C
Dppa5
Relative expression to GAPDH
d2
Basal
LIF
AICAR
AICAR+p38i
AICAR+LIF
AICAR+LIF+p38i
Dazl

**Figure S6. Analysis of Single-Cell RNA-Seq, Related to Figure 6**

(A) Pou5f1 (Oct4) expression. Left panel: t-SNE analysis. Purple dots indicate relative expression levels of each cell. Homogenous expression is observed in clusters 5, 6, 11, 12, and 15. Right panel: Violin plots. Higher expression is observed in clusters 5, 6, 11, 12, and 15.

(B) Violin plots for Nanog, Klf2 and Klf4.
(C) qPCR for Dppa5 and Dazl mRNA expression in Oct4GIP cells after 2 days culture with Basal, LIF alone, AICAR alone, AICAR+p38i, AICAR+LIF or AICAR+LIF+p38i condition. Expression levels are normalized to GAPDH. Data are represented as the mean ± SD (n=3; with technical triplicates).
