# Supplementary material for: AMPK activation reverts mouse epiblast stem cells to naive state
Source: iScience. 2021 Jun 25;24(7):102783. doi: 10.1016/j.isci.2021.102783 (PMC8283141; doi:10.1016/j.isci.2021.102783)
Supplement: Table S1. Components of reported protocols for naive reversion (mouse cells), related to discussion [file mmc2.docx]

Table S1. Components of reported protocols for Naïve reversion (mouse cells). Related to DISCUSSION

|  | Guo  (2009) | Hanna (2009) | Hall  (2009) | Silva (2009) | Guo (2010) | Gillich  (2012) | Tai  (2013) | Zhou (2010) | Ilich  (2016) | This  report |
| --- | --- | --- | --- | --- | --- | --- | --- | --- | --- | --- |
| Genetic induction | Klf4 | Klf4 | Klf2 | Nanog | Nr5a1/2 | Prdm14 | Gbx2 |  |  |  |
|  |  | c-Myc |  |  |  | Klf2 |  |  |  |  |
| Small molecules | GSK3i |  | GSK3i | GSK3i | GSK3i |  | GSK3i | GSK3i |  |  |
|  | MEKi |  | MEKi | MEKi | MEKi |  | MEKi | MEKi | (MEKi) |  |
|  |  | LIF | LIF | LIF | LIF | LIF | LIF |  |  | (LIF) |
|  |  |  |  |  |  |  |  | LSD1i |  |  |
|  |  |  |  |  |  |  |  | ALK5i |  |  |
|  |  |  |  |  |  |  |  | FGFRi |  |  |
|  |  |  |  |  |  |  |  |  | CK1αi |  |
|  |  |  |  |  |  |  |  |  |  | AMPK activator |
| Basement medium | N2B27 | N2B27 | N2B27 | N2B27 | N2B27 | DMEM+  20% KSR or N2B27 | N2B27 | DMEM+  20%KSR | DMEM+  20%KSR | GMEM+  10%KSR+  1%FBS |
| Chimeric mice | ○ | ○ | ○ | ○ | ○ | ○ | △^1^ | ○ | △^1^ | ○ |
| Germline transmission  mice | ○ | ○ | × | × | ○ | △^2^ | × | △^2^ | △^2^ | ○ |

( ): Small molecules which are not essential but promote reversion.

○: Chimera mice or mice through germline transmission were obtained using reverted cells.

×: Germline transmission experiment was not performed.

△^1^: Chimera embryos were confirmed but chimera mice were not checked.

△^2^: Gonad contribution was confirmed but mice in next generation were not checked.
