# Supplementary material for: AMPK activation reverts mouse epiblast stem cells to naive state
Source: iScience. 2021 Jun 25;24(7):102783. doi: 10.1016/j.isci.2021.102783 (PMC8283141; doi:10.1016/j.isci.2021.102783)
Supplement: Table S2. Primer sequences for qPCR, related to STAR Methods, Figure 2, S3, and S6 [file mmc3.docx]

Table S2. Primer Sequences for qPCR, Related to STAR Methods, Figure 2, S3, and S6

| Gene | Forward primer 5’-3’ | Reverse primer 5’-3’ |
| --- | --- | --- |
| GAPDH | TGTGTCCGTCGTGGATCTGA | TTGCTGTTGAAGTCGCAGGAG |
| Rex1 | TCTTCTCTCAATAGAGTGAGTGTGC | GCTTTCTTCTGTGTGCAGGA |
| Oct3/4 | GGACATGAAAGCCCTGCAGAA | GACAGATGGTGGTCTGGCTGAA |
| Nanog | GAATTCTGGGAACGCCTCATC | CCTTGTCAGCCTCAGGACTTG |
| Sox2 | AACCGATGCACCGCTACGA | TGCTGCGTAGGACATGCTG |
| Klf4 | AGACCAGATGCAGTCACAAGTC | CCCAGTCACAGTGGTAAGGTTT |
| Klf2 | CTAAAGGCGCATCTGCGTA | TAGTGGCGGGTAAGCTCGT |
| Esrrb | AGAGACCCTCTCCTATTCCCAC | TCTCCATGGGTTCCAGAAATGG |
| Tfcp2l1 | CCAGGAAAAGGCTAGAACCCAT | AGGTTCTCAGGTCTCCAGATCA |
| Dppa5 | CAGTCGCTGGTGCTGAAATA | TCCATTTAGCCCGAATCTTG |
| Dazl | TCGAAGGGCTATGGATTTGT | ACGTGGCTGCACATGATAAG |
